# Multi-Feature Classification of Displacement Alarm Causes Using IoT-Based Slope Monitoring Sensor Data

**DOI:** 10.3390/s26206378

**Published:** 2026-10-09

**Authors:** Sang-Yun Lee, Byung-Suk Park, Woo-Seok Kim, Sung-Pil Hwang

**Affiliations:** 1 Integrated Road Management Research Center, Korea Institute of Civil Engineering and Building Technology (KICT), Goyang-si 10223, Republic of Korea; sangyunlee@kict.re.kr; 2 Department of Geotechnical Engineering Research, Korea Institute of Civil Engineering and Building Technology (KICT), Goyang-si 10223, Republic of Korea

**Keywords:** slope monitoring, IoT sensor, displacement cause classification, clustering, feature extraction, multi-sensor analysis

## Abstract

Internet of Things (IoT)-based slope monitoring systems support real-time stability surveillance, but frequent false alarms cause alarm fatigue and delay responses to genuine hazards. We developed a clustering-based framework that classifies alarm causes into three categories—Malfunction, Actual Displacement, and Others—from inclinometer and extensometer data. From 55 alarm events recorded at 43 monitoring stations in 2025, 85 domain-informed features (statistical, waveform, spatial-correlation, trend, sudden-synchronization, and malfunction-pattern) were extracted and compared across five feature-extraction methods, six selection levels, and three clustering algorithms. Because the feature ranking uses expert-assigned labels, the framework is characterized as label-informed feature ranking followed by unsupervised clustering, and it was evaluated with 100 random seeds, nested leave-one-site-out validation, bootstrap confidence intervals, and permutation tests. A compact set of five features—three cross-correlation-based synchronization features and two spatial-correlation features—was stable across seeds and achieved a leave-one-site-out accuracy of 0.80 (95% CI 0.69–0.89) with the full 8 h window. Using only data recorded up to the alarm time, accuracy rose to 0.91 (0.84–0.98), 12 of the 13 Actual Displacement events were recovered (recall 0.92), and no non-displacement event was assigned to the displacement cluster. Synchronization features separated genuine displacement from the other causes, whereas spatial-correlation features distinguished external disturbance from sensor malfunction. The interpretable framework is proposed as a decision-support filter that complements conventional threshold-based alarms.

## 1. Introduction

Recent years have seen a steady increase in newly constructed slopes in Korea, while the management of aging slopes—many in service for more than 20 years—has emerged as a critical concern. At the same time, shifting rainfall patterns—driven by climate change—are intensifying slope instability. Hence, there is an urgent need for maintenance frameworks that focus on real-time, cause-aware monitoring, thereby moving beyond intermittent, judgment-based inspection.

Extreme weather events, driven by climate change, are fundamentally altering the rainfall patterns that trigger slope failures. During the 2011–2024 period, abnormal climate events accounted for approximately 79% of total disaster losses in Korea, with impacts extending across both marine and terrestrial environments [1]. The intensity of summer heavy rainfall has continued to rise: in July alone, rainfall-induced landslides affected 169 ha—nearly 95% of the total damaged area—revealing a pronounced temporal concentration of risk [2]. Such extreme rainfall rapidly increases pore-water pressure in the ground, reducing shear strength along potential failure surfaces and substantially elevating the risk of large-scale casualties and property damage [3,4].

Conventional slope management relies primarily on visual inspection and intermittent manual measurement. However, these approaches lack the temporal and spatial resolution needed to detect precursory signs of slope failures in real time [5,6]. Manual inspection is also constrained by inspector subjectivity, and access is frequently restricted by steep terrain and adverse weather—inherent structural limitations of human-dependent monitoring [6,7]. Consequently, the transition to Internet of Things (IoT)-based monitoring systems, capable of detecting minute slope movements and issuing alerts within minutes, has become a key national safety objective [8]. By continuously tracking subtle displacements, such systems provide the information needed for timely risk assessment and early warning.

In practice, however, field deployments are exposed to rainfall, temperature fluctuations, and biological interference (e.g., animals and vegetation). These challenges frequently result in data containing substantial noise and error [8,9,10]. Much of the prior work has therefore concentrated on filtering erroneous readings and extracting genuine displacement signals [9,10,11]. This filter-and-discard paradigm treats anomalous data primarily as unwanted and removes them. However, from an operational standpoint, why an alarm was triggered in the first place is the more pressing question. Continuous monitoring systems routinely generate false alarms because of sensor malfunction, communication failure, and biological interference, often inducing alarm fatigue among operators and delaying responses to genuine hazards. Therefore, improving slope disaster prevention requires a shift from the binary detection of displacement toward a systematic classification approach focusing on whether an alarm reflects actual ground displacement, a sensor malfunction, or another cause.

To address this gap, the present study classifies alarm causes into three categories—Malfunction, Actual Displacement, and Others—using inclinometer and extensometer data from an IoT-based slope monitoring system. Conventional alarm schemes only detect whether a displacement threshold has been exceeded, through a single absolute limit or a set of multi-level thresholds [12,13,14]. Hence, false alarms remain their principal operational drawback even after careful threshold calibration [15]. Therefore, the proposed approach involves identifying the underlying cause of each alarm. By distinguishing genuine displacement from sensor malfunction and other factors, this approach enables differentiated response strategies—immediate field inspection for true displacement and rapid maintenance for sensor-related faults.

Herein, a clustering-based approach is proposed to analyze the intrinsic patterns of the sensor signals. In the proposed framework, the expert-assigned event labels are used only to rank candidate features (label-informed feature ranking), whereas the partitioning of events is performed by unsupervised clustering that does not use the labels. Because the clustering step is label-free, the degree of agreement between the resulting clusters and the field labels serves as a check on whether the labeled categories correspond to distinct signal structures, and disagreement points to patterns not captured by the current labeling scheme. This is useful when labeled field data are scarce, although simple supervised classifiers trained on the same five features were found to reach a similar accuracy. Through the systematic combination of multiple feature-extraction and feature-selection techniques, the classification performance of alternative configurations was compared, and its stability was assessed by resampling. Further, the physical interpretability of the key features was examined to ground the resulting framework in domain-informed criteria.

From 55 event samples collected at 43 monitoring stations during field operations in 2025, 85 domain-informed features were extracted—spanning statistical, waveform, spatial-correlation, trend, sudden-synchronization, and malfunction-pattern groups—and evaluated across combinations of five feature-extraction methods and six feature-selection levels. Because the feature ranking uses the field labels, all performance figures are reported under a nested leave-one-site-out protocol with bootstrap confidence intervals in addition to the conventional full-data evaluation. A compact configuration of five features reproduced the field labels with a leave-one-site-out accuracy of 0.80 when the full 8 h window was used and 0.91 when only data available at the alarm time were used, recovering 12 of 13 Actual Displacement events without any false displacement classification. Cross-correlation-based temporal-synchronization features proved the most discriminative, while spatial-correlation features distinguished asynchronous event types. These results indicate that an interpretable classification framework, grounded in the relative temporal and spatial relationships among multiple sensors, can complement conventional alarm strategies based on absolute-value thresholds [12,14].

## 2. Theoretical Background

Effective development of a data-driven classification model requires a systematic understanding of slope displacement behavior, geotechnical and environmental factors inducing such behavior, and monitoring technologies for data acquisition. To establish the theoretical foundation, this section reviews continuous slope monitoring system configurations, defines main displacement types, examines key factors influencing slope stability, and introduces clustering techniques and feature selection methods for classifying displacement patterns from monitoring data.

### 2.1. Slope Monitoring System

**Figure 1:**
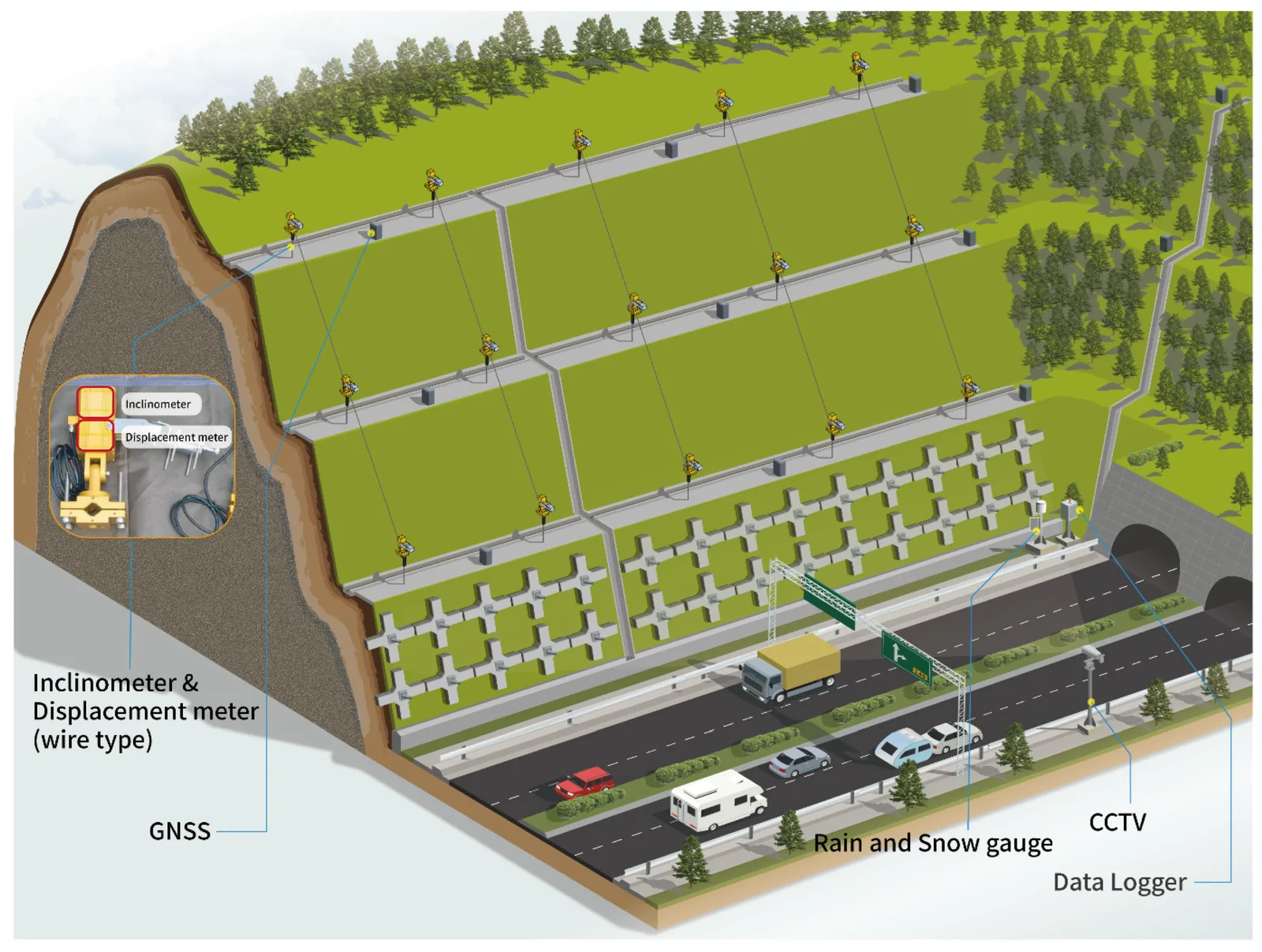
Schematic of the slope measurement system. CCTV, closed-circuit television; GNSS, global navigation satellite system.

A typical continuous slope monitoring system consists of an integrated framework comprising sensors, data acquisition and transmission devices, and a central analysis server designed to continuously assess slope stability (Figure 1). Data collected from field-installed sensors is converted into digital signals via data loggers and transmitted in real time to a central server via wired communication networks. The server stores and analyzes data to evaluate slope behavior and issue alerts upon detecting abnormal signals. From among the available continuous monitoring systems, inclinometers and extensometers, the data from which directly reflect slope behavior, were selected as primary analysis targets in this study.

Inclinometers measure horizontal ground displacement, enabling identification of internal deformation patterns and potential subsurface deformation zones. Extensometers quantitatively measure tensile and compressive deformation by tracking distance changes between two points. Using inclinometers and extensometers in combination provides complementary information regarding geometric characteristics and deformation magnitude, which a single sensor type may fail to capture. During actual ground displacement, temporally continuous and spatially consistent responses are typically observed across multiple sensors. In contrast, signals caused by sensor malfunction or external disturbances do not form coherent composite patterns. Therefore, integrated analysis of inclinometer and extensometer data provides a robust physical basis for distinguishing displacement events from non-displacement events.

In contrast, GNSS monitoring involves relatively high installation costs and provides only absolute displacement at individual points, limiting the feasibility of establishing a sufficiently dense network. Rain gauge data represent a triggering factor rather than a direct measurement of displacement.

Displacement is, however, the consequence of a deformation process governed by hydro-mechanical variables. Rainfall infiltration reduces matric suction (negative pore-water pressure) and raises the pore-water pressure within the slope, and these changes can precede any displacement that is large enough to be detected by inclinometers or extensometers. Direct measurement of soil suction therefore offers a physically earlier indicator than displacement alone. Field measurement of suction has long been limited by cavitation and by the slow response of conventional tensiometers, but recent developments such as osmotic tensiometers capable of measuring high suction and following rapid wetting–drying transitions in long-term deployments [16] make continuous suction monitoring increasingly feasible. Integrating suction, pore-water pressure, and soil-moisture sensors with inclinometers, extensometers, and rain gauges in an IoT network would provide a stronger physical basis for early warning than displacement thresholds alone. The monitoring network analyzed in this study does not include suction or pore-water pressure sensors. Rainfall is recorded at each station, but the number of events accumulated so far is too small to assess its contribution to cause classification reliably. Accordingly, this study focuses on the inter-sensor relationships derived from inclinometer and extensometer data to establish a data-driven classification framework for identifying the causes of displacement alarms; the integration of rainfall and hydro-mechanical measurements is left for future work.

### 2.2. Main Displacement Types

Continuous monitoring systems are exposed to environmental conditions like weather changes, biological activity, and communication interruptions. Therefore, monitoring data often contains various types of noise in addition to signals generated by slope displacement. In previous studies, threshold-based filtering and statistical outlier removal techniques have been applied to differentiate erroneous data from genuine displacement signals.

Jakopec et al. [17] calculated Z-scores for displacement vector components derived from photogrammetry and applied the three-sigma rule to classify vectors outside the expected range as outliers. In a study on filtering high-frequency GNSS landslide displacement data, Sharifi et al. [18] noted that the mean and standard deviation can be distorted by extreme outliers, adopting a median-based Hampel filter to address this limitation. Within a moving window (running bandwidth), the median of displacement measurements was computed, and three times the median absolute deviation (MAD) was set as the threshold to remove data outside this range. While previous studies have focused on filtering erroneous data through statistical outlier removal, such approaches may misinterpret rapidly occurring rockfall events as errors or fail to capture subtle precursory displacements.

**Figure 2:**
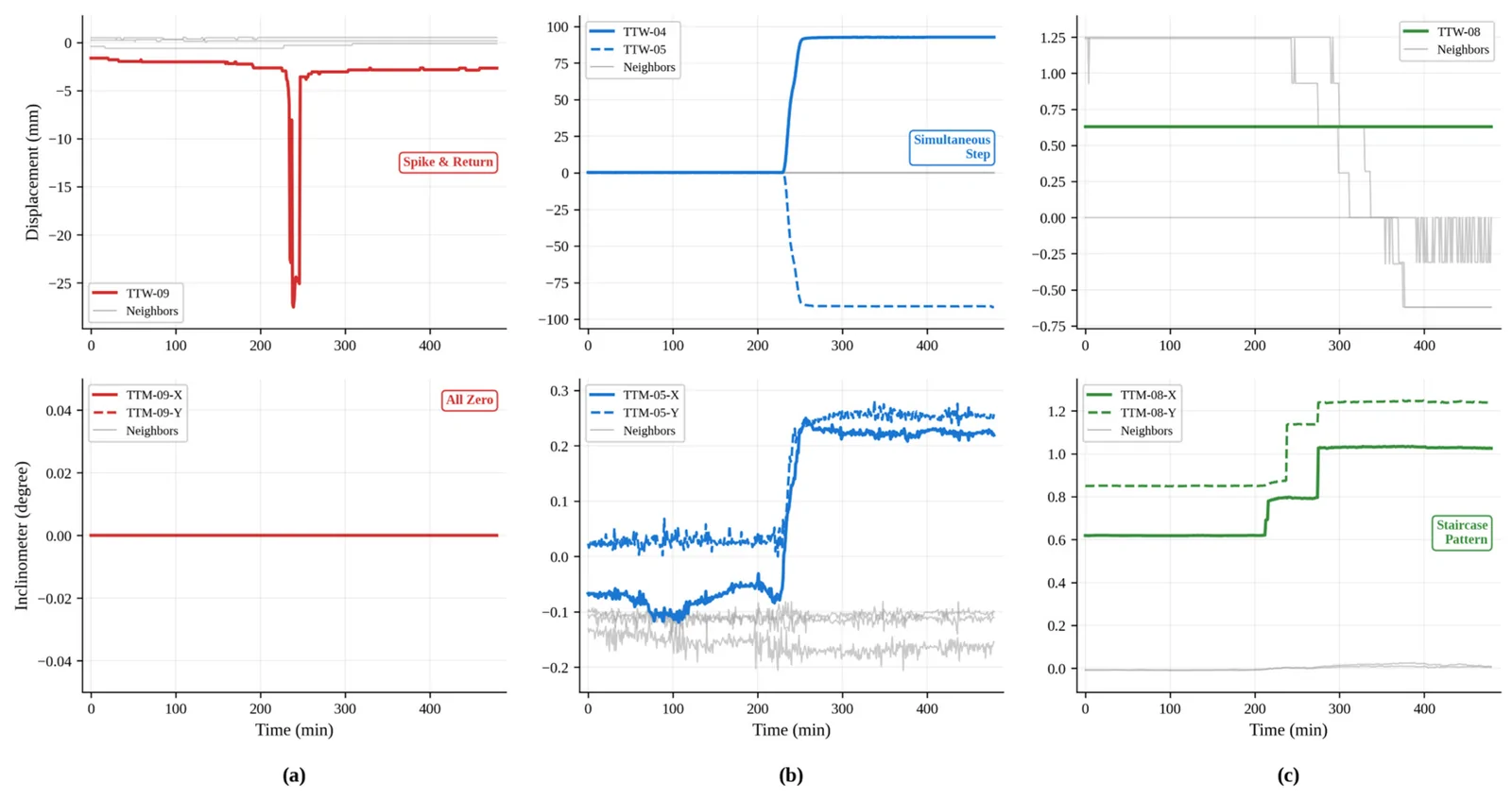
Examples of data labeling: (**a**) Malfunction, (**b**) Actual displacement, and (**c**) Others. Top row, extensometer displacement; bottom row, inclinometer tilt; gray lines, neighboring sensors. In (**a**), the inclinometer readings of TTM-09 and its neighboring sensors are all 0 and therefore overlap.

**Figure 3:**
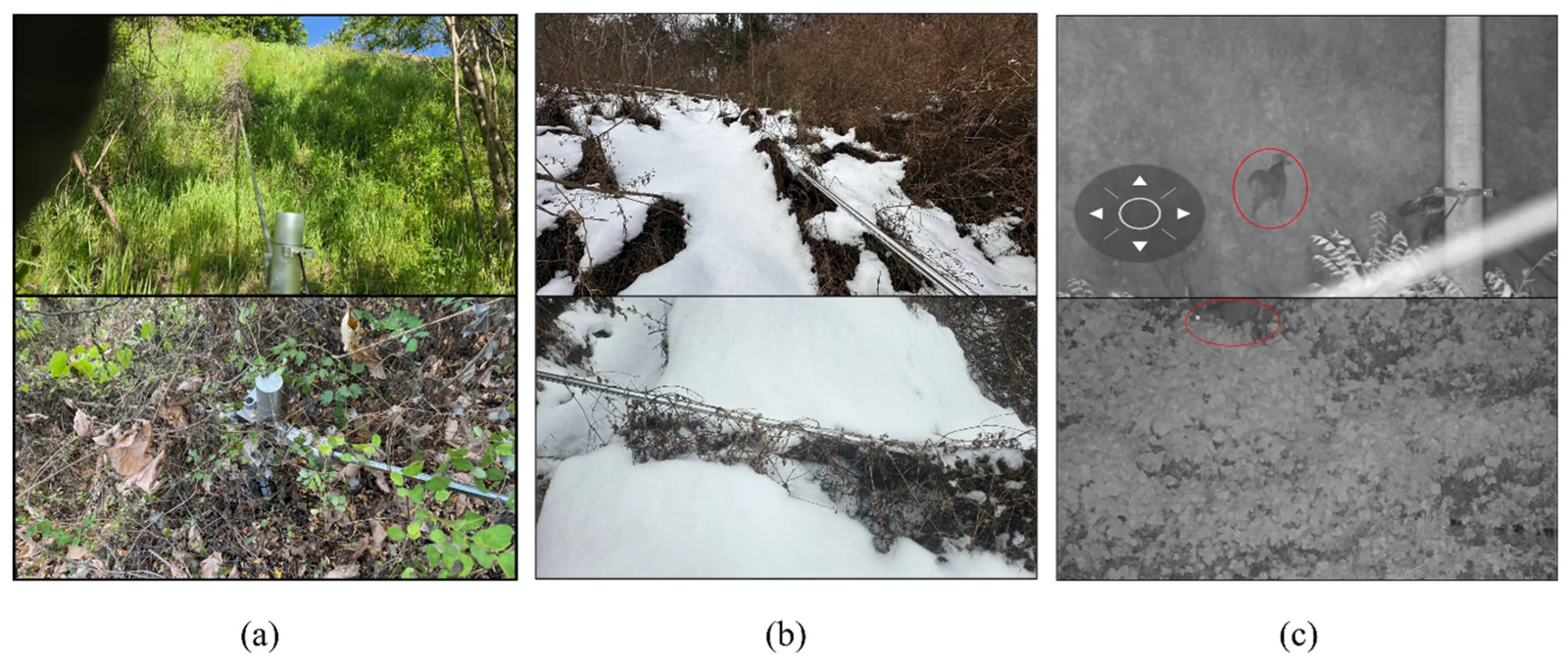
Field examples of alarms classified as Others: (**a**) vines wrapping around a sensor; (**b**) snow accumulated on a sensor; (**c**) a wild animal in contact with a sensor. Red circles in (**c**) indicate the wild animals captured in the CCTV images.

The IoT-based slope monitoring system developed by the Korea Institute of Civil Engineering and Building Technology (KICT) collects and stores raw monitoring data without prior filtering and applies alarm management criteria based on unprocessed data. Consequently, false alarms arising from multiple causes occur frequently. To address this issue, monitoring data have been systematically labeled. Currently, events are categorized into four types: Malfunction (sensor malfunction or communication failure), Inspection (sensor replacement and routine inspection), Actual Displacement (rockfall, soil sliding, or tree overturning), and Others (alarms caused by an external physical disturbance of the instrument rather than by ground displacement, a sensor or communication fault, or a scheduled inspection—typically vines wrapping around a sensor, snow accumulating on and loading a sensor, or wild animals touching a sensor as they move past). Representative signal patterns for each type are illustrated in Figure 2. Field photographs of representative Others cases are shown in Figure 3.

### 2.3. Clustering

Clustering is a representative unsupervised learning technique that groups data points with similar characteristics based on intrinsic structure without predefined labels [19,20,21,22]. Unlike supervised learning, which learns input–output relationships using labeled data, clustering is primarily applied in exploratory data analysis to identify latent patterns and structural relationships within data.

The adoption of clustering in this study was motivated by several considerations. First, labeling performed by field experts involves subjective judgment; partitioning the events by an algorithm that does not itself use the labels—the labels influence only the choice of features—provides a check of whether the labeled categories correspond to separable signal patterns. Second, because clustering groups events by their signal characteristics alone, it can reveal natural divisions of the data that do not coincide with the operational categories—for instance, a coarser split between synchronous and asynchronous events—and thus indicates where the label scheme is well supported by the signals and where it is not. Third, clustering enhances the interpretability of the classification criteria by quantitatively deriving the key inter-sensor relationships contributing to displacement cause classification. It should be stressed, however, that the three-class structure is not the dominant source of variance in the full feature space; the approach therefore depends on the label-informed selection of features and is not an unsupervised method in the strict sense.

Both internal and external metrics were used to evaluate clustering quality. Internal metrics assess cluster cohesion and separation without reference to ground truth labels. The silhouette score, Calinski–Harabasz index, and Davies–Bouldin index were used. Conversely, external metrics quantify agreement between clustering results and actual labels. The adjusted Rand index (ARI), normalized mutual information (NMI), and purity were calculated for this purpose.

#### 2.3.1. K-Means

K-means is a centroid-based partitioning clustering algorithm that divides a dataset into a predefined number of k clusters [19,23,24]. The algorithm iteratively minimizes the within-cluster sum of squares, defined as the sum of squared Euclidean distances between each data point and the centroid of its assigned cluster.

In the k-means procedure, k initial centroids are first selected. Each data point is assigned to the nearest centroid. Subsequently, centroids are recomputed as the mean of data points assigned to each cluster. The assignment and update steps are repeated until centroid convergence according to a predefined stopping criterion.

K-means offers high computational efficiency and ease of implementation, making it suitable for large-scale datasets. However, it requires prior specification of the number of clusters k and assumes spherical cluster distributions, which may limit performance when data exhibit non-spherical structures. In this study, the number of clusters was set to n_clusters = 3 to correspond to the three displacement cause categories (i.e., Malfunction, Actual Displacement, and Others). To mitigate variability from random centroid initialization, the algorithm was executed with n_init = 10, and the solution with the lowest within-cluster sum of squares was retained.

#### 2.3.2. Hierarchical Clustering

Hierarchical clustering is an algorithm that constructs a hierarchical structure based on similarities among data points [25]. This method enables visualization of cluster merging or splitting processes through a dendrogram, facilitating intuitive analysis of overall data structure and cluster formation.

Hierarchical clustering can be categorized into agglomerative and divisive approaches. In this study, the agglomerative approach was adopted because of its computational efficiency and suitability for the dataset scale. The bottom-up strategy progressively captures fine-grained local similarity patterns among sensor displacement signals, aligning with the objective of identifying distinct displacement behavior types from individual sensor measurements. Agglomerative hierarchical clustering initially treats each data point as an individual cluster. Inter-cluster distances are computed, and the two most similar clusters are iteratively merged. This process continues until the predefined number of clusters is reached.

Various linkage methods compute inter-cluster distances, including Single Linkage, Complete Linkage, Average Linkage, and Ward Linkage. In this study, the Ward Linkage method was applied. Ward Linkage minimizes the increase in within-cluster variance resulting from cluster merging, tending to produce clusters of relatively balanced size.

A key advantage of hierarchical clustering is that the number of clusters need not be predetermined, as the dendrogram allows exploration of cluster structures at multiple levels. However, computational complexity generally increases to O(n^2^) or higher, limiting applicability to large datasets. Because the dataset used in this study consisted of 55 event samples, hierarchical clustering was feasible. A comparative analysis with k-means examined differences in classification performance across clustering algorithms.

#### 2.3.3. Density-Based Spatial Clustering of Applications with Noise (DBSCAN)

DBSCAN is a density-based clustering algorithm that forms clusters according to density distribution in data space [20,26,27]. The algorithm defines clusters as regions of high point density and classifies points in low-density regions as noise. Unlike k-means, DBSCAN does not require the predefinition of the number of clusters and can detect clusters with arbitrary shapes.

DBSCAN operates based on two key parameters: eps and min_samples. The parameter eps defines the radius within which neighboring points are considered. The parameter min_samples specifies the minimum number of neighboring points required to define a core point. If a point has at least min_samples neighbors within its eps radius, it is classified as a core point.

The algorithm proceeds as follows. An unvisited data point is selected, and the number of neighbors within its eps radius is computed. If this number is at least min_samples, the point is designated as a core point, and a new cluster is created, incorporating its neighboring points. If any neighboring points are also core points, their neighbors are recursively expanded into the cluster. If the number of neighbors is less than min_samples, the point is classified as a border point or noise. This process continues until all data points are examined.

The primary advantages of DBSCAN include automatic determination of cluster number and natural identification of outliers as noise. It effectively detects clusters with non-spherical shapes, providing flexibility across diverse data distributions. However, performance is sensitive to the selection of eps and min_samples, and accuracy may degrade when clusters of varying densities coexist. In this study, eps was estimated as the 90th percentile of the k-nearest neighbor distance distribution, and min_samples was set to 3.

### 2.4. Feature Selection Methods

Feature selection identifies a subset of features from the original feature set that meaningfully contribute to classification or clustering performance [28]. For high-dimensional data, using all features may degrade model performance due to the curse of dimensionality and increase overfitting risk caused by irrelevant or noisy features.

The primary objectives of feature selection are improving model generalization, enhancing computational efficiency, and increasing interpretability. Eliminating noisy features improves classification performance on unseen data. Reducing the number of features lowers computational cost during training and inference. Focusing on core features also enables intuitive interpretation of key factors contributing to classification results.

Feature selection methods are generally categorized into filter, wrapper, and embedded approaches. Filter methods select features based on statistical characteristics independent of model training and are computationally efficient. Wrapper methods directly evaluate model performance to determine optimal feature combinations, often achieving high accuracy at substantial computational cost. Embedded methods perform feature selection during model training.

In this study, considering computational efficiency and interpretability, a filter-based approach using the Between-class Variance (BCV) Ratio was adopted as the feature importance metric. The BCV Ratio is the ratio of the between-class variance to the total variance of each feature (the proportion of variance explained by the class labels, η^2^), and therefore lies between 0 and 1. Higher values indicate stronger discriminative contribution between event types. Based on the derived feature importance ranking, the top 5, 10, 15, 20, 25, and 30 features were sequentially selected. Clustering performance was evaluated for each configuration to determine the optimal feature number.

## 3. Materials and Methods

This section presents the methodology for classifying displacement causes based on slope monitoring data. The overall analytical framework is described, including data structure, sensor configuration, event selection criteria, feature extraction, feature selection techniques, and clustering-based experimental design.

### 3.1. Data Structure

**Figure 4:**
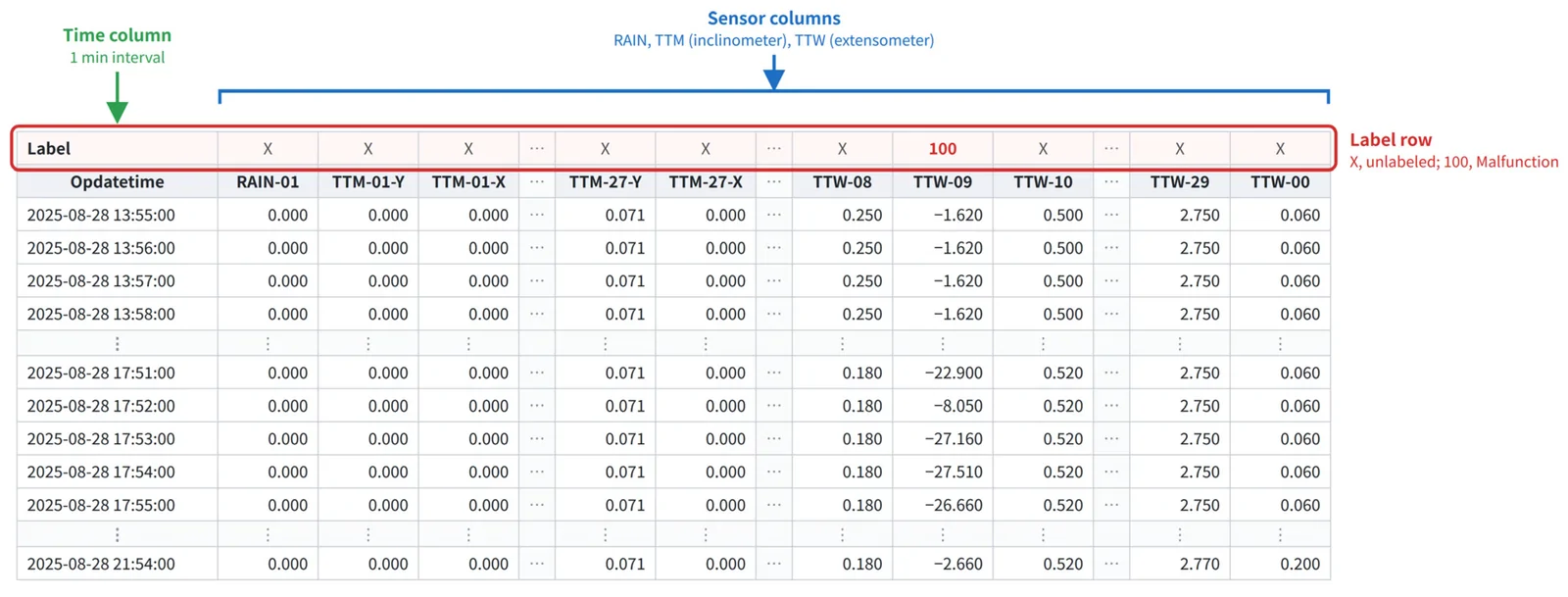
Sensor data file structure. Excerpt of one raw data file, in which ellipses indicate omitted columns and rows. The red box marks the label row (X, unlabeled; 100, Malfunction), the green arrow the time column, and the blue arrow the sensor columns.

The dataset used in this study consists of sensor monitoring data collected from the IoT-based slope monitoring system. Each CSV file is structured as shown in Figure 4. The label row (red box) contains the event label (Malfunction, Inspection, Actual Displacement, Others, or Unlabeled) assigned to each column. Below the label row, the table consists of a time column (denoted by the green arrow), recording the measurement timestamp at 1 min intervals, and multiple sensor columns (denoted by the blue arrow), containing the corresponding sensor measurement values.

The clustering analysis focused on three categories: Malfunction, Actual Displacement, and Others. The Inspection label, corresponding to alarms generated during routine maintenance procedures, was excluded because such events can be operationally identified in advance and are expected to be removed through planned improvements in field inspection procedures, where sensors will be temporarily removed from the monitoring system prior to scheduled inspections. The final dataset consisted of 55 samples, including 30 Malfunction, 13 Actual Displacement, and 12 Others events, recorded at 43 monitoring stations distributed across ten provinces and metropolitan regions of Korea (Chungnam, Chungbuk, Jeonbuk, Jeonnam, Gyeongbuk, Gyeongnam, Gangwon, Gyeonggi, Sejong, and Ulsan); the number of displacement sensors per station ranged from 17 to 112. The categories follow the operational definitions introduced earlier; for the Others class, representative field photographs of the disturbances involved (vines, snow loading and wild animals) are shown in Figure 3.

### 3.2. Sensor Configuration

The sensors used in this study were categorized as composite and standalone sensors. The monitoring network integrates sensors supplied by several domestic manufacturers, and all records are collected by the monitoring system in a common format at 1 min intervals; the proposed method uses only these standardized records and does not depend on a specific instrument model.

Composite sensors comprised an inclinometer and extensometer integrated within the same unit and were designated TTM and TTW. TTM-##-X and TTM-##-Y measured tilt along the X- and Y-axis directions, respectively. TTW-## was a wire-based extensometer measuring horizontal displacement. Sensors sharing the same identification number (e.g., TTM-01-X, TTM-01-Y, and TTW-01) were installed at the same physical location and were therefore used for spatial correlation analysis.

Standalone sensors consisted of individually installed inclinometers (TM) and extensometers (TW), positioned independently from composite sensors. A rain gauge (RAIN) was also installed at every station; however, the rainfall record was not used in the present analysis because the number of events accumulated so far is insufficient to evaluate its contribution reliably, and all features were derived exclusively from the displacement sensor data.

### 3.3. Event Detection Criteria

To detect meaningful events from sensor time-series data, three event detection algorithms were applied.

**Figure 5:**
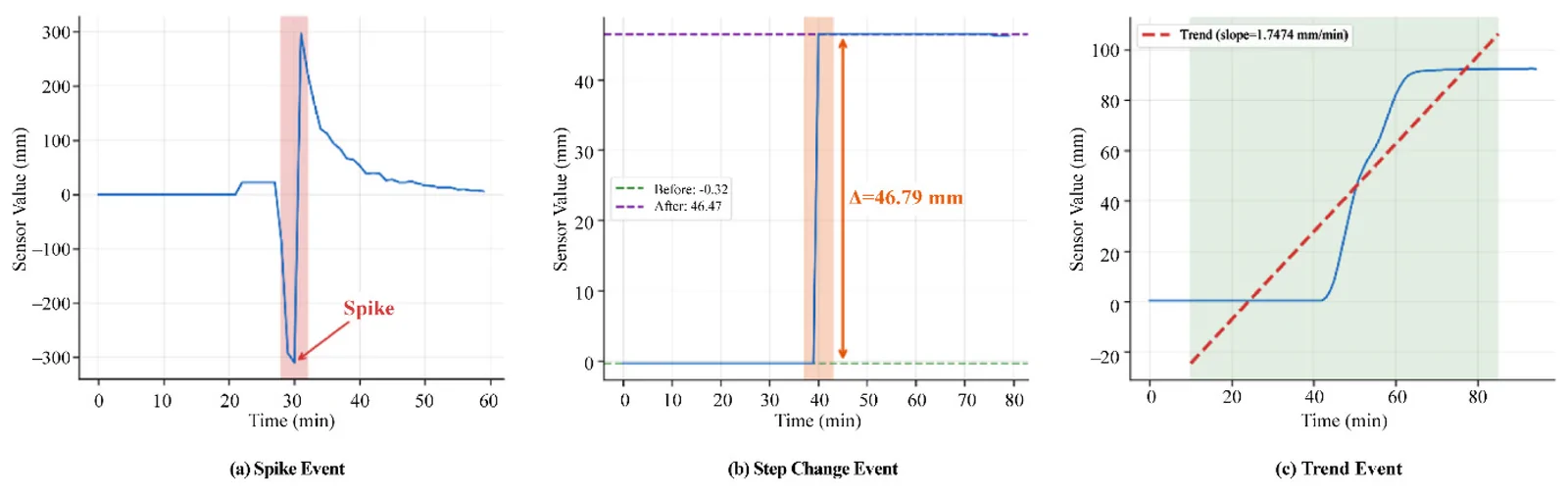
Example of event patterns: (**a**) spike event; (**b**) step change event; (**c**) trend event. Blue lines indicate the sensor readings. In (**a**,**b**), the shaded bands mark ±2 min around the detected spike and ±3 min around the detected change point, respectively, and in (**b**), the green and purple dashed lines indicate the mean values before and after the change point. In (**c**), the green band marks the segment detected as a trend, and the red dashed line is the least-squares linear fit over this segment.

A Spike Event detects abrupt changes exceeding the mean of first-order differences plus three times the standard deviation. These events are primarily attributed to sensor malfunctions or external shocks. A Step Change identifies statistically significant permanent level shifts by comparing mean values before and after a 30 min window using a *t*-test. Step changes represent a core characteristic of Actual Displacement events. A Trend Event detects gradual changes by performing linear regression over multi-scale sliding windows of 20, 40, and 100 min. A trend is identified when the coefficient of determination R^2^ ≥ 0.3 and the slope exceeds an adaptive threshold. Overlapping trends are merged based on reliability criteria. An example of event patterns is shown in Figure 5.

### 3.4. Feature Extraction

Five feature extraction methods were applied to each sample. Structural dataset attributes such as total length and number of sensors were excluded because all samples share uniform data length, making this attribute non-discriminative, and sensor count describes the station rather than the displacement behavior itself.

Method 1 (Statistical + Waveform) extracted 33 features combining basic statistical metrics, including event count, magnitude, duration, and rate of change, with waveform characteristics such as timing, shape, and cross-correlation. Method 2 (Spatial + Trend) extracted 21 features combining spatial characteristics, including inter-sensor correlations at the same location and propagation properties, with trend characteristics such as count, duration, magnitude change, and linearity. Method 3 (Hybrid + Trend) integrated Methods 1 and 2, resulting in 54 features. Method 4 (Hybrid + Trend + Step) extended Method 3 by adding step-change features, including step count, magnitude, confidence level, and simultaneous multi-sensor level shifts, yielding 60 features. Method 5 (All Features) further extended Method 4 by incorporating sudden synchronization features such as sse_num_events and sse_event_coverage, as well as malfunction-pattern features such as mf_inclinometer_malfunction_ratio. This method produced a comprehensive set of 85 features.

### 3.5. Feature Selection

The candidate feature set was deliberately broad: every feature hypothesized to reflect a difference between the three alarm causes was included, so that the importance of each could be evaluated on the same basis rather than pre-judged. Because weakly discriminative features add noise to the distances on which k-means operates, only the highest-ranked features were carried forward to the clustering step. For each feature, the BCV Ratio—the ratio of the between-class variance to the total variance, computed using the expert-assigned event labels—was calculated as an importance score, and, based on the resulting ranking, the top 5, 10, 15, 20, 25, and 30 features were selected for the clustering experiments.

Feature importance therefore reflects the degree to which each feature separates the three event types; features with large between-class and small within-class variance yield higher scores. It should be emphasized that this ranking step is supervised (label-informed): the class labels enter the analysis here, and only the subsequent clustering step is unsupervised. Consequently, the agreement between clusters and labels is an optimistic estimate when the ranking is computed on the full dataset. To quantify this effect, the ranking was repeated inside a leave-one-site-out validation loop in which the held-out station did not contribute to the ranking, and the results were compared with label-free feature selection methods.

### 3.6. Clustering Experimental Design

Missing observations in the raw 1 min series (short communication gaps) were handled inside each feature extractor: they were excluded from the computation of the statistical, synchronization, spatial, and malfunction-pattern features and forward/backward-filled for the step-change detector, and correlation coefficients that were undefined were set to 0. The resulting 55 × 85 feature matrix contained no missing or infinite values, and the features were standardized with StandardScaler before clustering. Experiments combined five feature extraction methods, six feature selection levels (top 5, 10, 15, 20, 25, and 30), and three clustering algorithms.

For k-means, the number of clusters was set to k = 3 to match the three operational alarm categories; the appropriateness of this choice with respect to the intrinsic structure of the data was additionally examined using silhouette, gap-statistic, and stability criteria. Each k-means run used ten random centroid initializations (n_init = 10), and the solution with the lowest within-cluster sum of squares (inertia) was retained; this criterion does not use the class labels. Because the results of k-means depend on the random seed, every experiment was repeated over 100 seeds, and performance is reported as a distribution (mean ± SD) rather than as the best single value. Hierarchical clustering was implemented using Ward Linkage with k = 3. For DBSCAN, eps was estimated as the 90th percentile of the k-nearest neighbor distance distribution, and min_samples was set to 3. Evaluation metrics included internal metrics (silhouette score, Calinski–Harabasz index, and Davies–Bouldin index) and external metrics (ARI, NMI, and purity). All analyses were performed in Python 3.14.1 (Python Software Foundation, Wilmington, DE, USA) using NumPy 2.3.5, pandas 2.3.3, SciPy 1.17.0, and scikit-learn 1.8.0; figures were produced with Matplotlib 3.10.8.

## 4. Results

**Figure 6:**
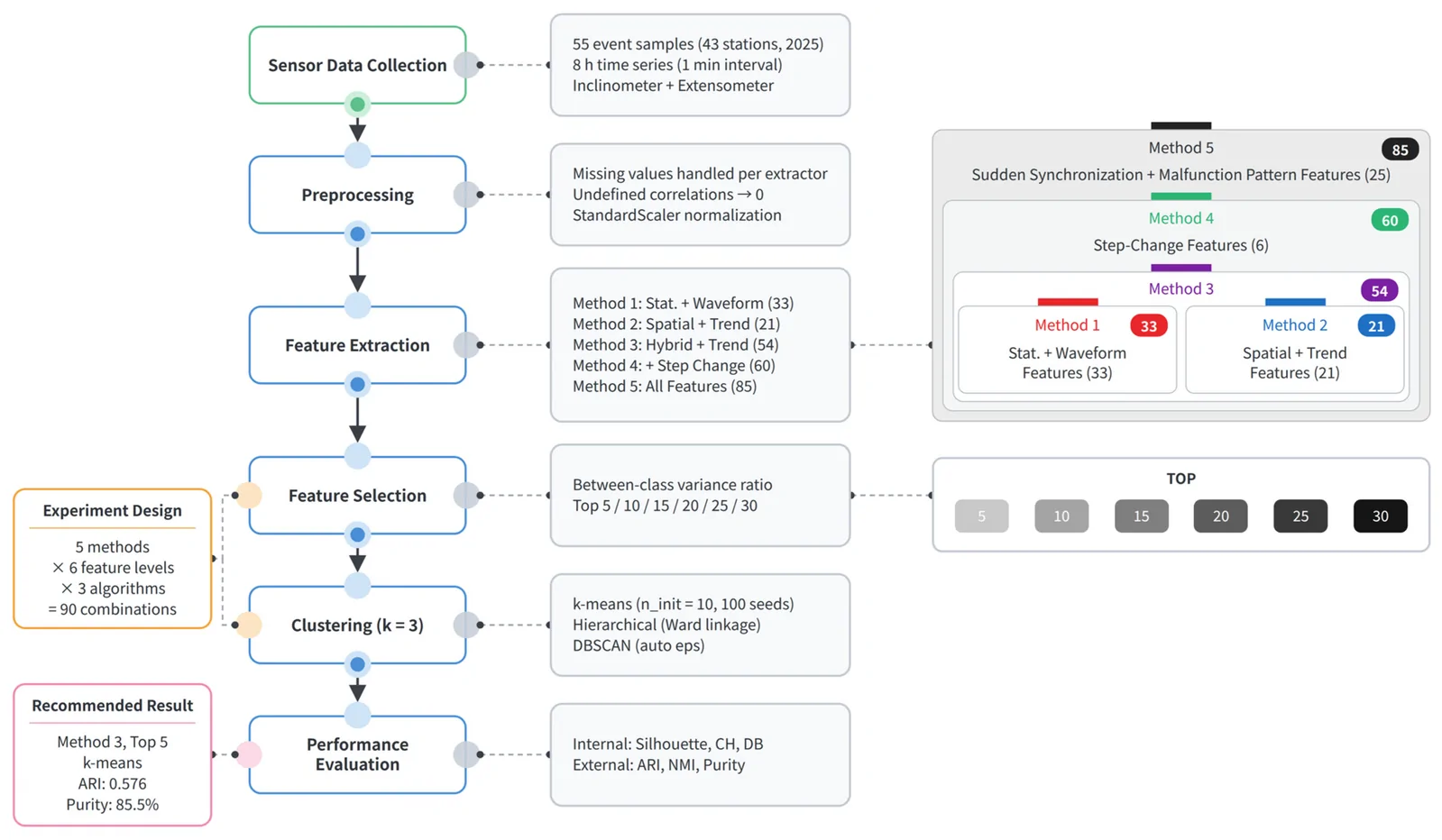
Data clustering flowchart. Solid arrows indicate the processing sequence, and dashed lines connect each step to its settings; colored labels distinguish the five feature extraction methods (number of features in boxes), and gray-scale boxes indicate the numbers of top-ranked features evaluated. CH, Calinski–Harabasz index; DB, Davies–Bouldin index; ARI, adjusted Rand index; NMI, normalized mutual information; DBSCAN, density-based spatial clustering of applications with noise.

This section presents results of clustering-based displacement cause classification experiments conducted using five feature extraction methods and feature selection techniques. The overall analytical workflow is illustrated in Figure 6.

### 4.1. Dataset Composition

The dataset consisted of 55 event samples collected in 2025 from slope ground displacement monitoring sensors. Each sample comprised 8 h of 1 min time-series data centered on the alarm time recorded by the monitoring system, i.e., 4 h before and 4 h after the alarm. The alarm time was used as the reference time t0 of every sample, and the features were additionally recomputed from windows truncated at t0 to assess how much of the classification depends on post-alarm data. Events were classified into three cause categories based on expert interpretation (Table 1).

Malfunction events accounted for 54.5% of the dataset, representing the largest proportion, while Actual Displacement and Others accounted for 23.6% and 21.8%, respectively. Malfunction events made up more than 80% of the labeled alarms in the raw dataset; to limit class imbalance, 30 of them were drawn at random so that the three classes were represented in a ratio of roughly 2:1:1, while all available Actual Displacement and Others events were retained. Even after this subsampling, Malfunction remains the largest class, reflecting operational monitoring environments in which sensor malfunctions occur far more often than genuine displacement.

### 4.2. Feature Extraction and Selection Results

#### 4.2.1. Number of Features by Extraction Method

To capture diverse characteristics of sensor signals, five feature extraction methods were designed. Only sensor-signal-based features were used in the analysis, excluding six label-related features and four basic information features (Table 2).

#### 4.2.2. Feature Importance Ranking

**Figure 7:**
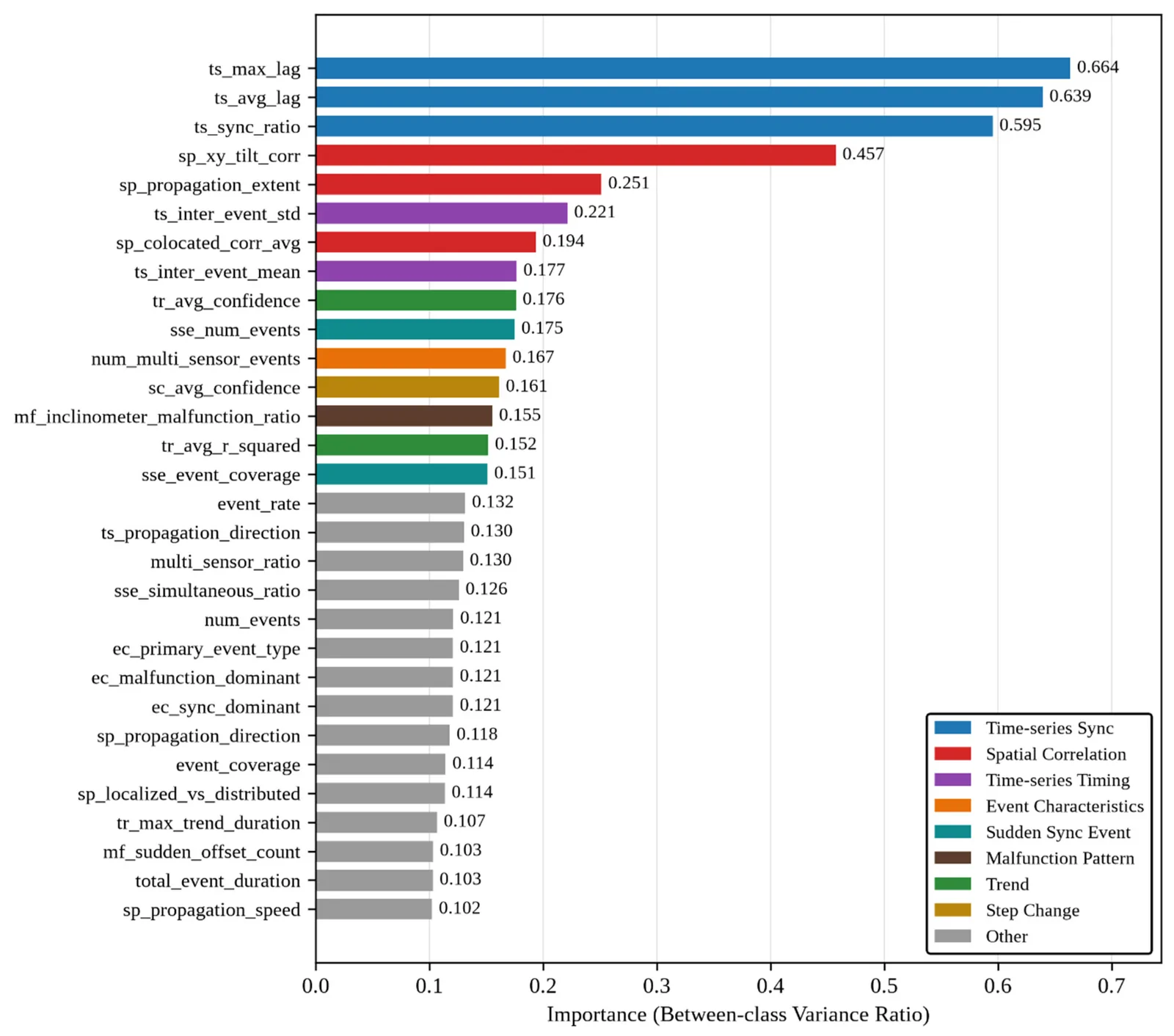
Feature importance ranking of the top 30 features by BCV Ratio (based on Method 5); bar colors indicate the feature category of the top 15 features.

Features with strong class-separating capability were identified using the BCV Ratio. This metric represents the ratio of the between-class variance to the total variance of a feature, where higher values indicate greater discriminative power among event types. Because Method 5 incorporates all features from the other methods, it was selected as the basis for feature importance analysis. The top 15 features derived from Method 5 are presented in Table 3 and Figure 7.

Cross-correlation-based time-series synchronization features ranked in the top three positions, while spatial correlation features ranked fourth and fifth, confirming complementary contributions of temporal and spatial characteristics. In addition, the newly introduced sudden-synchronization features (ranks 10 and 15) and the malfunction-pattern feature (rank 13) of Method 5 entered the top 15, although with importance scores (0.15–0.18) far below those of the synchronization features; whether they actually improve the clustering was therefore tested separately by a controlled ablation.

### 4.3. Analysis of Feature Selection Results

Building on the feature importance ranking established above, this section describes how different combinations of feature extraction methods, feature subset sizes, and clustering algorithms affect overall classification performance.

#### 4.3.1. Cross-Method Performance Comparison

**Figure 8:**
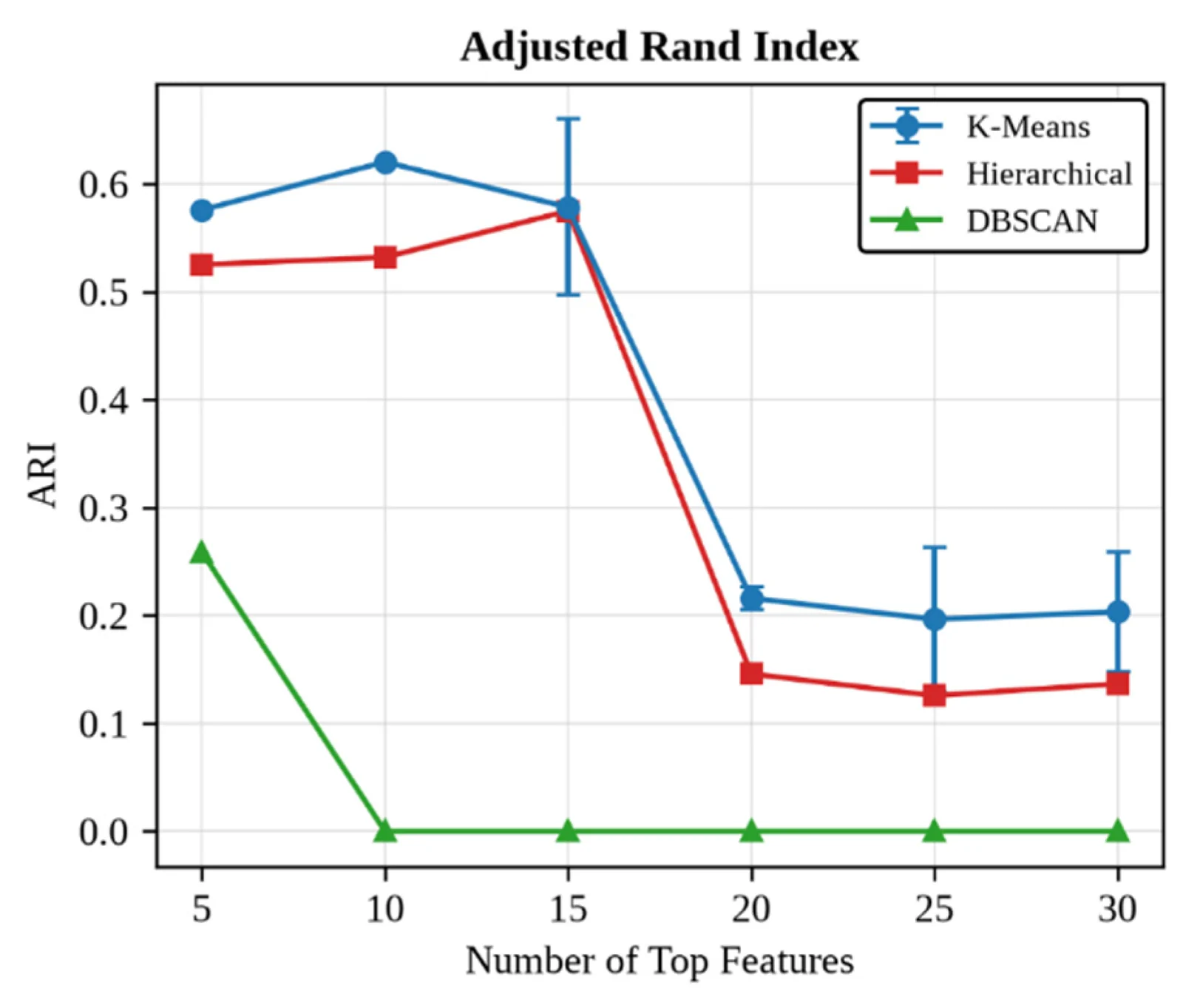
ARI by number of selected features for the three clustering algorithms (Method 5; k-means shown as mean ± SD over 100 random seeds). ARI, adjusted Rand index; DBSCAN, density-based spatial clustering of applications with noise; SD, standard deviation.

**Figure 9:**
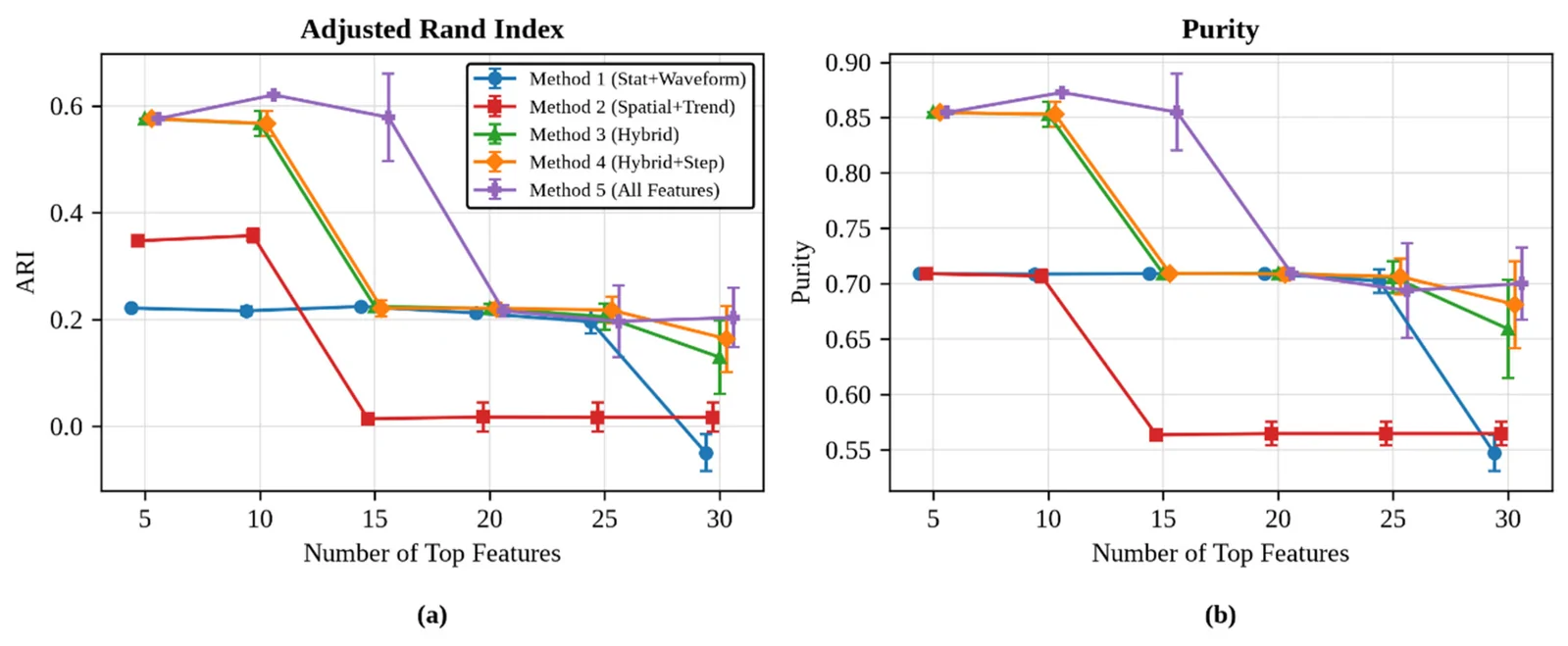
Clustering performance by number of selected features for the five feature extraction methods (k-means; mean ± SD over 100 random seeds): (**a**) ARI; (**b**) purity. ARI, adjusted Rand index; SD, standard deviation.

To examine the impact of feature quantity on clustering performance, experiments were conducted using the top 5, 10, 15, 20, 25, and 30 ranked features for each method. Figure 8 presents performance trends across the three clustering algorithms under Method 5, while Figure 9 compares performance across the five extraction methods under k-means. The main experimental results are summarized in Table 4; for k-means, Table 4 and Figure 8 and Figure 9 report the mean and standard deviation over 100 random seeds rather than a single run.

The analysis yielded the following findings.

First, Methods 1 and 2 alone reached ARI values of only 0.22 and 0.35, whereas Method 3, which integrates Method 1 (Statistical + Waveform) and Method 2 (Spatial + Trend), reached 0.576 with five features, confirming the complementary contributions of temporal and spatial characteristics.

Second, the best configurations were compact and stable. With the top 5 features, Methods 3, 4, and 5 selected the same five features (ts_max_lag, ts_avg_lag, ts_sync_ratio, sp_xy_tilt_corr and sp_propagation_extent) and gave identical results in all 100 seeds (ARI 0.576, purity 85.5%, silhouette 0.452). Method 5 with the top 10 features gave a slightly higher ARI (0.621, purity 87.3%) that was also seed-independent, whereas the top-15 configuration of Method 5 was strongly seed-dependent (ARI 0.579 ± 0.082; its maximum of 0.669 occurred in only 3% of the seeds), so that a single run of this configuration could give a misleadingly high value. Seed-averaged values are therefore used throughout, and the five-feature configuration is adopted as the recommended configuration for the reasons given below.

Third, excessive feature inclusion led to performance degradation. For Methods 1–4, ARI fell to about 0.2 or below once more than 10 features were included, and Method 5 followed at 20 features (e.g., Method 2 from 0.357 at top 10 to 0.014 at top 15; Method 3 from 0.567 at top 10 to 0.224 at top 15). With 55 events, the additional lower-ranked features add noise that dominates the Euclidean distances used by k-means—the practical manifestation of the curse of dimensionality for a candidate set of 85 features.

#### 4.3.2. Recommended Feature Configuration

Because missing a genuine displacement is the most serious error in operation, the primary criterion for selecting the recommended configuration was the recall of Actual Displacement under nested validation, subject to no non-displacement event being assigned to the displacement cluster; overall agreement with the field labels, parsimony, and seed stability were used as secondary criteria. On this basis, the five-feature configuration (Method 3, top 5) is adopted as the recommended configuration in the remainder of the paper. With the data available at the alarm time, it recovers 12 of the 13 Actual Displacement events without a false displacement assignment—more than any other of the 30 method/feature-number combinations—and it is also the most compact configuration, identical for Methods 3–5 on the full data and independent of the random seed. With the full 8 h window, no configuration recovers more than 9 of the 13 events, and the ten-feature configurations that reach 9 lose this advantage at the alarm time. Performance of the three clustering algorithms under this configuration is summarized in Table 5.

K-means gave the highest agreement with the field labels and hierarchical clustering was slightly lower, whereas DBSCAN found only two density-based groups, essentially separating the highly synchronized Actual Displacement events from all other events.

**Figure 10:**
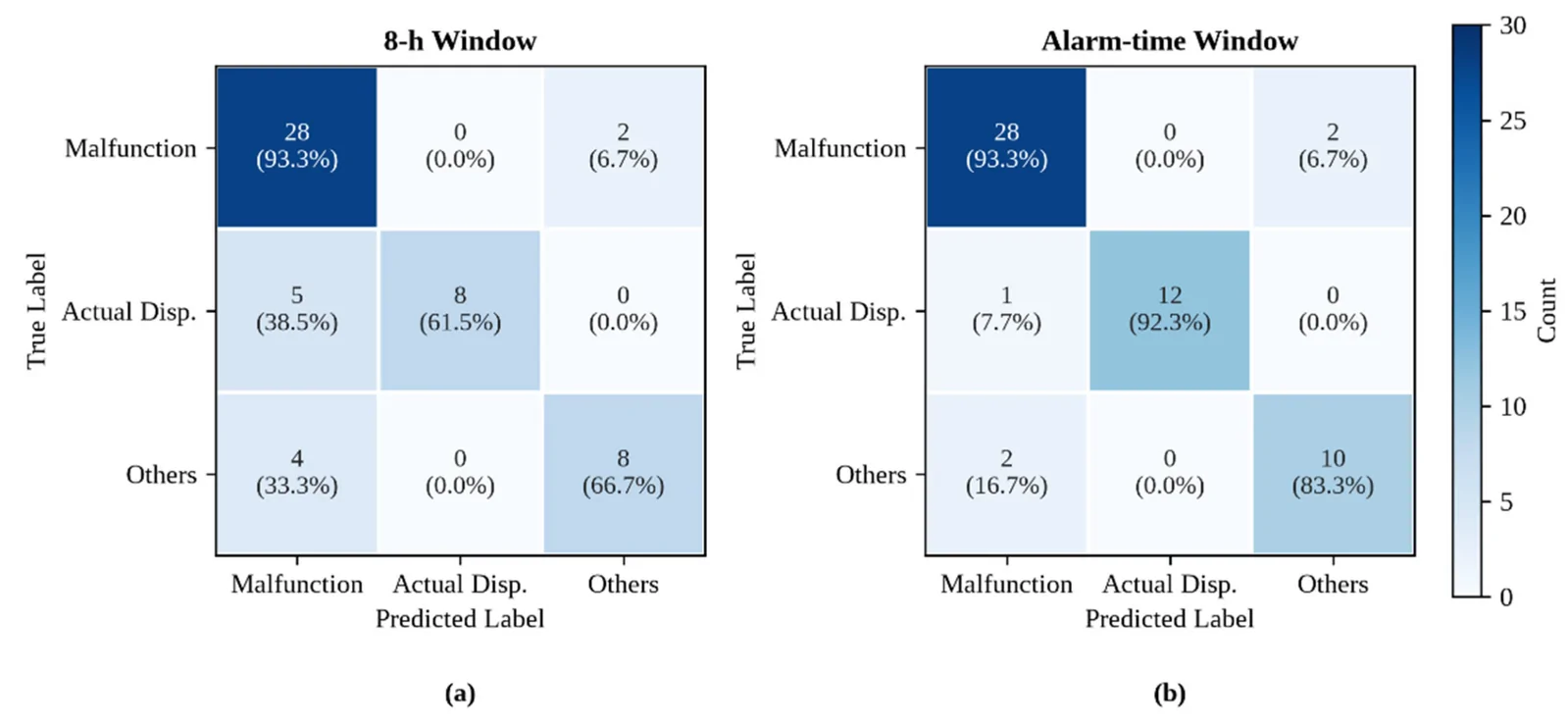
Confusion matrices for the recommended configuration (Method 3, top 5, k-means) under nested leave-one-site-out validation: (**a**) full 8 h window; (**b**) alarm-time window [t0 − 4 h, t0].

**Figure 11:**
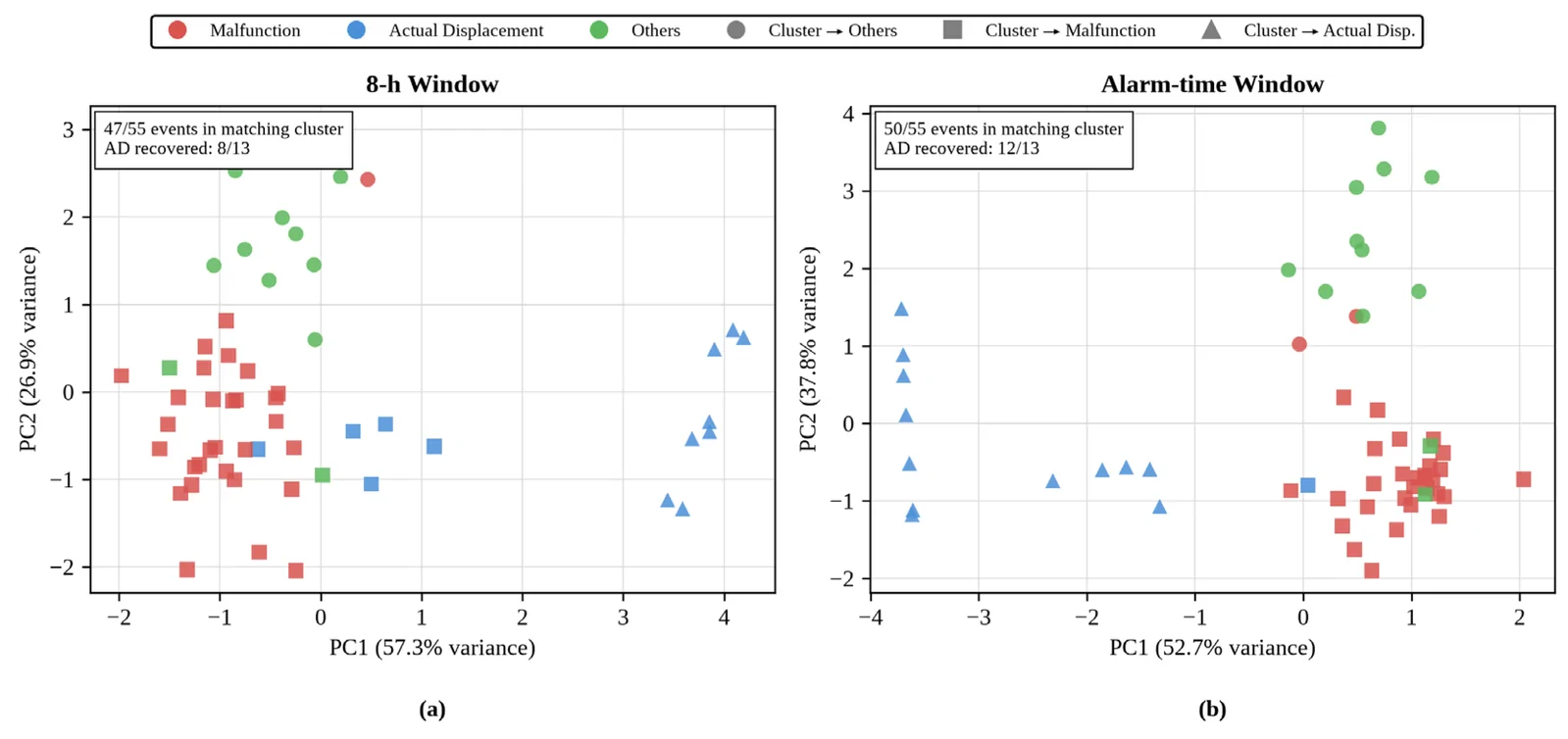
PCA-based 2D visualization of the five recommended features: (**a**) 8 h window; (**b**) alarm-time window. Colors indicate the field label, and marker shapes indicate the k-means cluster (mapped to its majority class).

**Figure 12:**
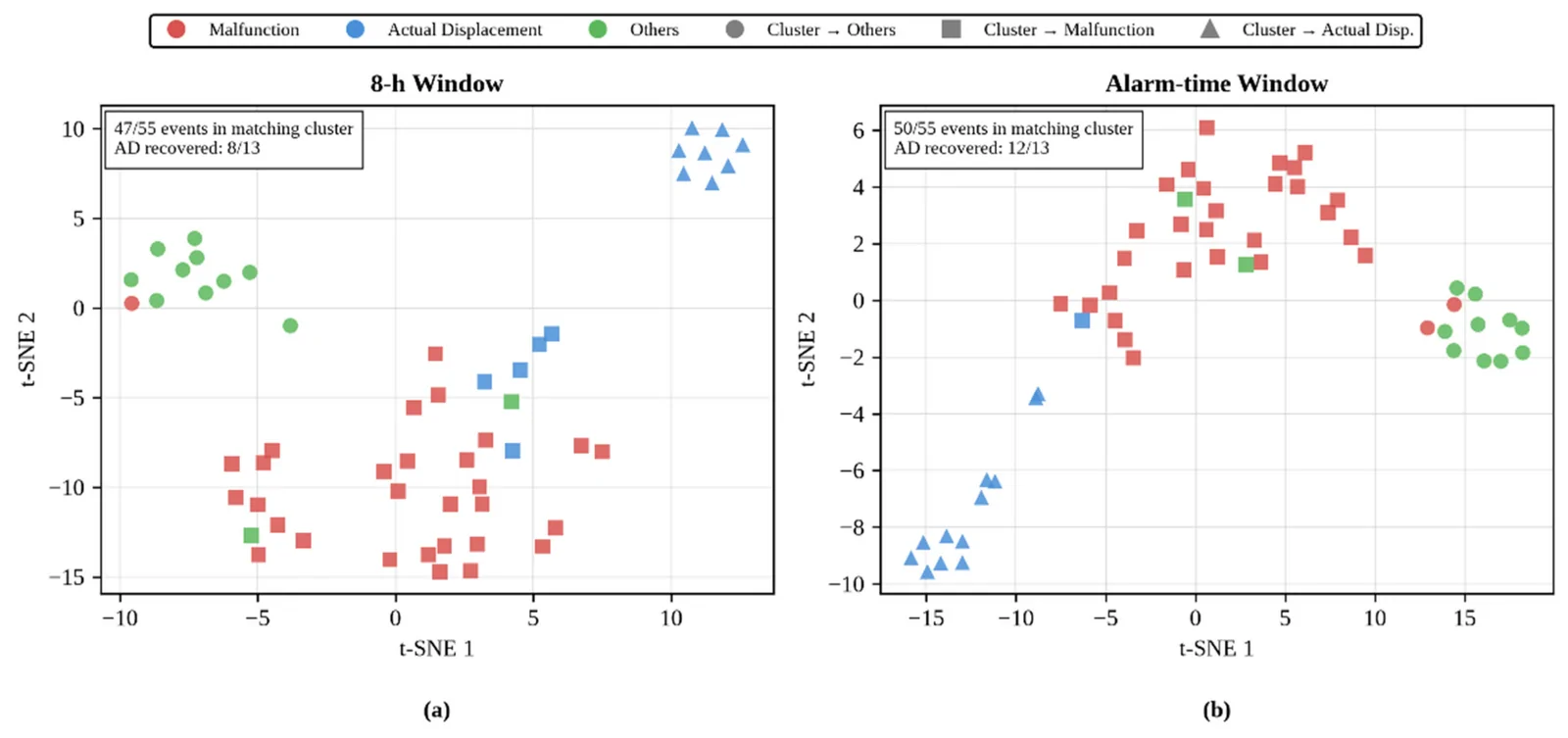
t-SNE-based 2D visualization of the five recommended features: (**a**) 8 h window; (**b**) alarm-time window. Colors indicate the field label, and marker shapes indicate the k-means cluster (mapped to its majority class).

Under this configuration, 47 of the 55 events (85.5%) fell into the cluster matching their field label; class-specific results and the confusion matrices obtained under nested validation are presented in Figure 10. Two-dimensional (2D) visualizations based on PCA and t-SNE of the five selected features are presented in Figure 11 and Figure 12 for the 8 h window (panels a) and for the alarm-time window (panels b). With the 8 h window, the highly synchronized Actual Displacement events form a distinct group, whereas the remaining five Actual Displacement events lie within the Malfunction region, and the Malfunction and Others clusters partially overlap; the silhouette score of 0.452 indicates moderate rather than strong intrinsic separation. With the alarm-time window, 12 of the 13 Actual Displacement events separate from all other events, and only one remains in the Malfunction region.

### 4.4. Physical Interpretation of Key Features and Event-Type Characteristics

**Figure 13:**
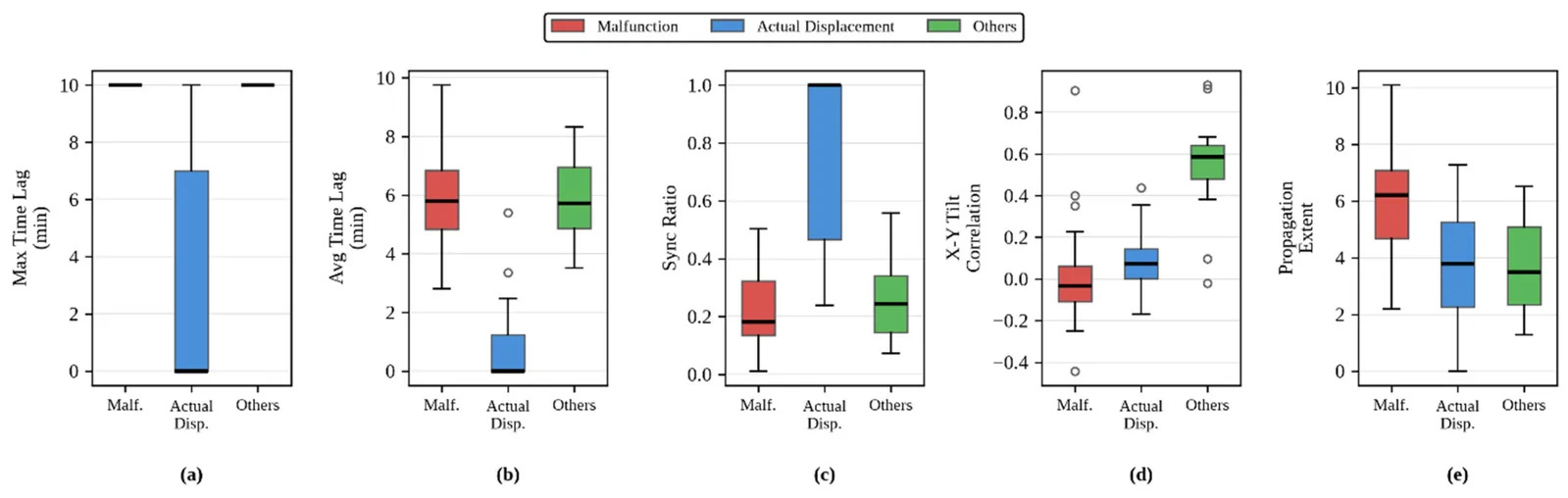
Distribution of key features by event type: (**a**) ts_max_lag; (**b**) ts_avg_lag; (**c**) ts_sync_ratio; (**d**) sp_xy_tilt_corr; (**e**) sp_propagation_extent. Boxes span the interquartile range, black lines indicate the median, whiskers extend to the most extreme values within 1.5 times the interquartile range, and open circles indicate outliers.

Table 6 presents descriptive statistics of key features by event type. Box plots in Figure 13 visually compare feature distributions across the three event categories.

#### 4.4.1. Time-Series Synchronization Features

The top three features are cross-correlation-based metrics, indicating that inter-sensor time-lag patterns constitute a primary factor in displacement cause classification. In Actual Displacement events, ts_max_lag was typically less than 10 min, reflecting the rapid propagation of physical displacement signals. The ts_sync_ratio exhibited a high mean synchronization ratio of 0.77 in Actual Displacement events, whereas Malfunction (0.22) and Others (0.25) showed substantially lower values (Table 6); however, five Actual Displacement events at densely instrumented stations had ratios between 0.24 and 0.50 and were the events missed by the clustering. These time-series features primarily separate Actual Displacement events from non-displacement events.

#### 4.4.2. Spatial Correlation Features

The feature sp_xy_tilt_corr enables fine-grained differentiation between asynchronous event types: Malfunction and Others. Higher values observed in Others events indicate that the X- and Y-axis tilts of the affected inclinometers change simultaneously, as expected when a unidirectional external force acts on the sensor pole or its mounting (e.g., vines wrapping around the sensor, snow loading, or contact by wild animals; Figure 3). In contrast, Malfunction and Actual Displacement events exhibit lower correlation values due to random noise and complex three-dimensional deformation, respectively. The feature sp_propagation_extent showed the widest propagation extent in Malfunction events, representing failure types that affect the overall monitoring system.

#### 4.4.3. Event Characteristics, Sudden Synchronization, and Trend Features

The features ts_inter_event_std and ts_inter_event_mean represent regularity and frequency of event occurrences. The feature num_multi_sensor_events reflects overall sensor network activity, and sc_avg_confidence reflects the confidence of the detected level shifts. Among newly introduced features in Method 5, sse_num_events indicates that repeated sudden synchronization occurs most frequently in Others events. The feature mf_inclinometer_malfunction_ratio shows the highest sensitivity for detecting inclinometer malfunction patterns in Others events. The feature tr_avg_confidence exhibits highest values in Others events, reflecting the consistent, monotonic drift that results when an external physical disturbance displaces a sensor, which then remains in its new position, in contrast to the erratic fluctuations characteristic of Malfunction events.

#### 4.4.4. Feature-Based Classification Rules

Physical interpretation of key features and statistical characteristics by event type was used to derive classification rules, as summarized in Table 7.

The framework follows a two-stage structure. First, Actual Displacement events are identified using synchronization ratio and time-lag metrics. Second, among asynchronous events, sp_xy_tilt_corr is used to distinguish Others from Malfunction.

Applied directly to the 55 events, the rules of Table 7 classify 45 events (81.8%) correctly with the 8 h features (recall: Actual Displacement 8 of 13, Malfunction 27 of 30, Others 10 of 12) and 48 (87.3%) with the alarm-time features (9 of 13, 29 of 30 and 10 of 12); no non-displacement event satisfies the displacement rule in either case. The k-means partition recovers more displacement events at the alarm time (12 of 13) because its boundary adapts to the alarm-time feature distribution, whereas the thresholds of Table 7 were derived from the 8 h distributions of Table 6 and should be re-derived from alarm-time data before operational use.

#### 4.4.5. Feature Correlation Analysis

**Figure 14:**
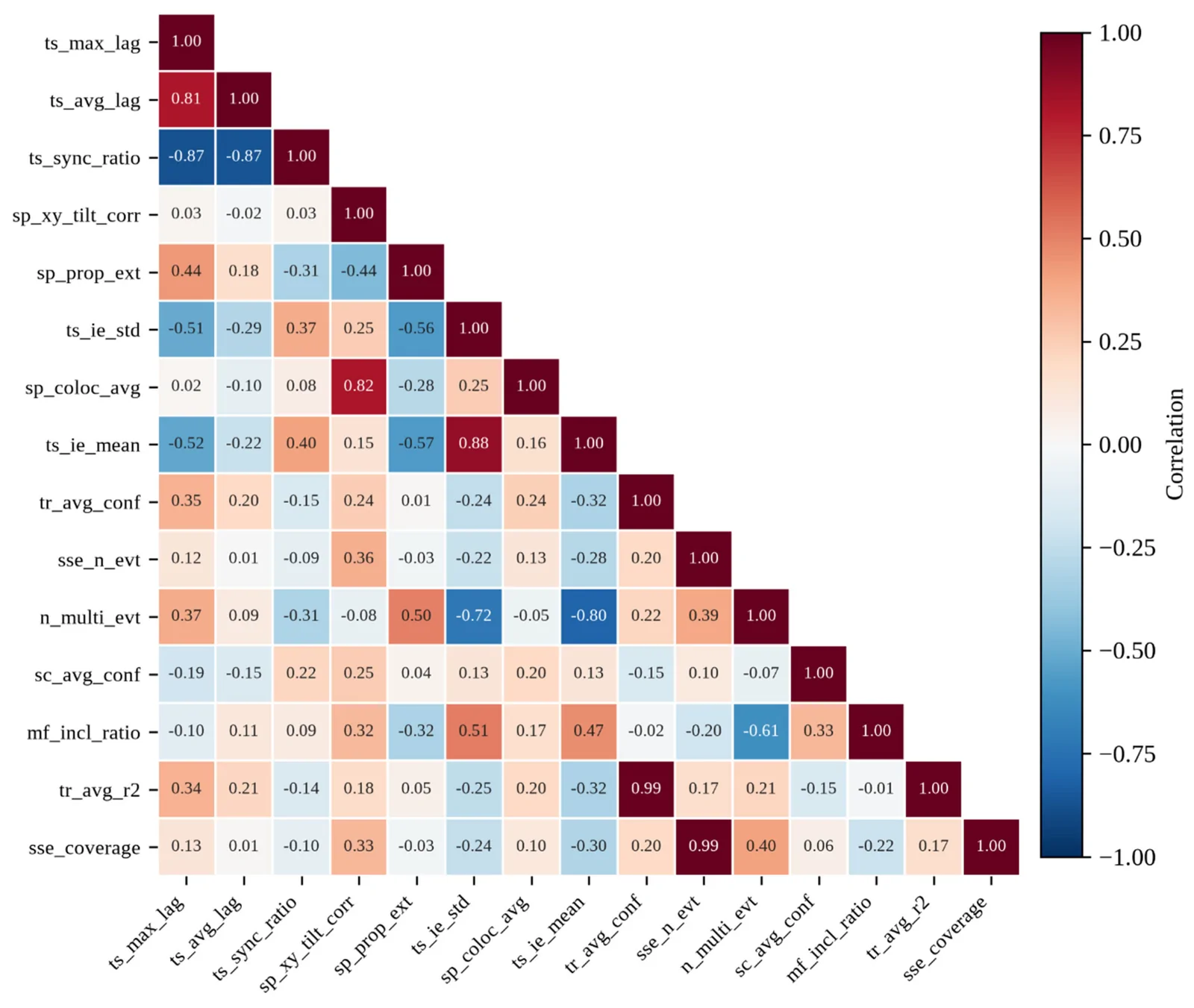
Feature correlation heatmap analysis results (Method 5, top 15).

The feature correlation heatmap shown in Figure 14 indicates strong correlation exceeding 0.9 between ts_max_lag and ts_avg_lag, and moderate negative correlation between ts_sync_ratio and ts_avg_lag. In contrast, correlations between time-series synchronization features and spatial correlation features are below 0.3, indicating that the two feature groups capture largely independent information. Sudden synchronization and malfunction-pattern features also demonstrate partial independence from the existing features; however, this independence did not translate into a consistent performance gain once seed dependence and label leakage were controlled.

### 4.5. Validation and Stability Analysis

The results presented so far were obtained by ranking the features on the complete dataset and then clustering the same events. Because the BCV ranking uses the field labels, this evaluation is optimistic. This section quantifies (i) the dependence of the results on the random seed, (ii) the performance obtained when ranking, scaling, and clustering are all fitted without the evaluated events, (iii) the performance of label-free feature selection, (iv) the contribution of the detector features of Method 5, and (v) class-specific performance. Unless stated otherwise, the analyses use the full 8 h window; the alarm-time window is treated separately afterwards.

#### 4.5.1. Dependence on the Random Seed

Figure 9 shows ARI and purity of k-means as a function of the number of selected features for the five methods, expressed as mean ± SD over 100 random seeds. The top-5 configurations of Methods 3–5 were identical in every seed (SD = 0), and Method 5 with the top 10 features was equally stable. In contrast, the top-15 configuration of Method 5 varied between seeds (SD = 0.082) because the additional, weakly discriminative features create several near-equivalent local optima of the k-means objective. Selecting the seed with the highest ARI would use the labels a second time and inflate the apparent performance; all values reported in this paper are therefore seed averages.

#### 4.5.2. Nested Leave-One-Site-Out Validation

To remove the label information shared between feature selection and evaluation, the complete pipeline—standardization, BCV ranking, selection of the top-N features, re-standardization and k-means—was fitted on the events of 42 stations and applied to the events of the held-out station (leave-one-site-out, LOSO; 43 folds). Held-out events were assigned to the nearest training centroid, and the cluster-to-class mapping was determined on the training fold only by Hungarian matching. The same procedure was repeated with single events held out (leave-one-event-out, LOEO; 55 folds) and for 20 random seeds. Uncertainty was quantified by bootstrapping the pooled held-out predictions (1000 resamples of events), and the statistical significance of the full-data pipeline was assessed by a permutation test in which the labels were permuted 1000 times and the complete label-informed pipeline was re-run on each permutation.

**Figure 15:**
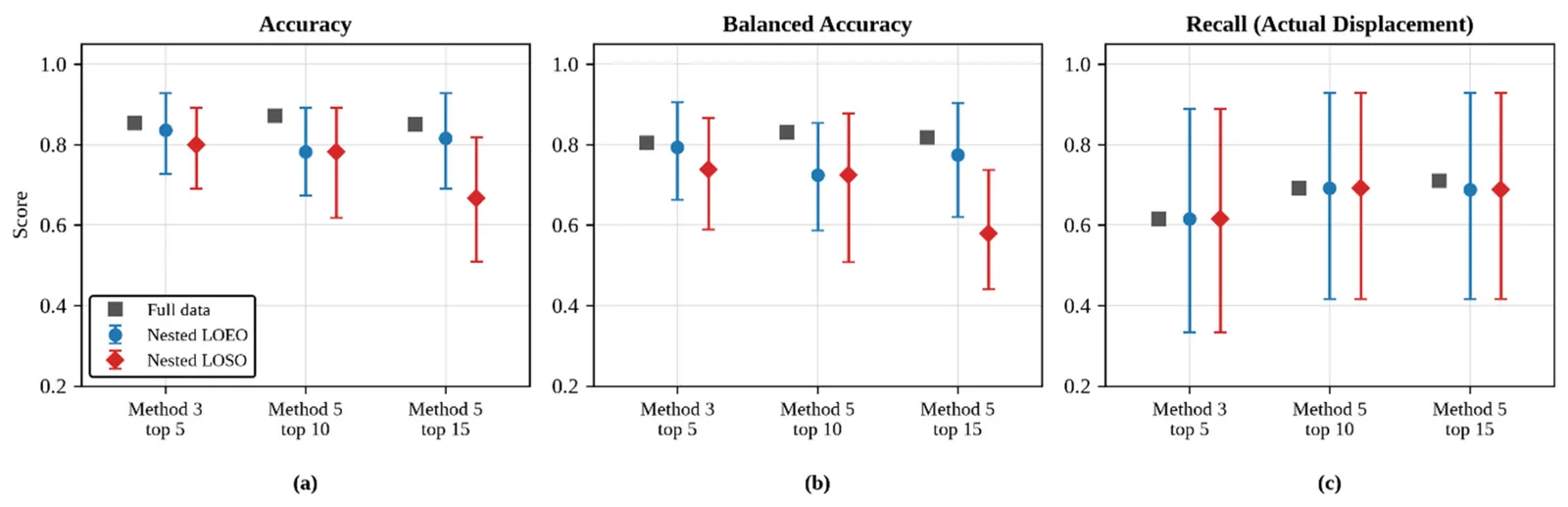
(**a**) Accuracy, (**b**) balanced accuracy, and (**c**) recall of Actual Displacement for three configurations under the full-data evaluation and under nested leave-one-event-out (LOEO) and leave-one-site-out (LOSO) validation (8 h window; bars are 95% bootstrap confidence intervals). Squares, circles, and diamonds indicate the full-data evaluation, nested LOEO, and nested LOSO, respectively.

Table 8 and Figure 15 compare the full-data evaluation with the nested estimates. For the recommended configuration, the LOSO accuracy was 0.80 (95% CI 0.69–0.89), balanced accuracy 0.74 (0.59–0.87), macro-F1 0.77 (0.62–0.88), Cohen’s κ 0.64 and ARI 0.44 (0.23–0.68), compared with 0.855, 0.805, 0.837, 0.743, and 0.576 in the full-data evaluation. The difference between the two evaluations measures the optimism introduced by the label-informed ranking: about five percentage points of accuracy for the five-feature configuration, but 19 percentage points for the Method 5 top-15 configuration (0.852 → 0.667), whose recall for the Others class collapsed from 0.82 to 0.21 when the station was held out. The larger feature set had therefore partly learned station-specific rather than cause-specific structure—seven of the twelve Others events originate from a single station—which is a second reason for preferring the compact configuration. The permutation test showed that the agreement obtained by the full-data pipeline is far above chance: with permuted labels the same label-informed pipeline produced a mean ARI of 0.05 (95th percentile 0.17, maximum 0.33 in 1000 permutations) against the observed 0.576 (*p* = 0.001, i.e., none of the 1000 permutations reached the observed value). The label-informed ranking therefore does not by itself explain the recovered class structure, although it does inflate the point estimates.

Because the labels enter the pipeline twice—in the feature ranking and in the cluster-to-class mapping—the nested estimate is effectively that of a label-guided nearest-centroid classifier, and it is natural to ask how a conventional supervised classifier would perform on the same five features. Linear discriminant analysis (LDA) and L2-regularized logistic regression were therefore trained inside the same LOSO loop (feature ranking on the training fold, top 5 features). With the 8 h window, logistic regression reached the same accuracy as k-means (0.800; balanced accuracy 0.715, ARI 0.480) with a higher recall of Actual Displacement (11 of 13 vs. 8 of 13) but a lower recall of Others (0.33), and LDA was slightly lower (0.764); with the alarm-time window both classifiers were identical to k-means (accuracy 0.909, 12 of 13 displacement events recovered). The clustering step thus performs on a par with simple supervised classifiers on this dataset. Its role is therefore not to outperform a supervised classifier but to show that the labeled categories correspond to separable groups in a feature space whose partition was not fitted to the labels—which is what makes the agreement informative about the label scheme itself—and to provide the interpretable classification rules of Table 7. For an operational module, a supervised classifier or a hybrid that uses the cluster structure to set a conservative displacement boundary may be preferable, particularly with respect to the recall of Actual Displacement. Two further caveats apply to the nested estimates. First, the recommended configuration was chosen among the 30 method/feature-number combinations partly on the basis of its nested performance, so a small selection optimism remains in the reported values; the choice is, however, also supported by a priori criteria (parsimony, seed independence and identity across Methods 3–5). Second, the station contributing seven Others events was classified as a single held-out fold: five of its seven events were recovered, and the recall of Others at the remaining stations was 3 of 5, so the Others cluster is not merely a station signature.

#### 4.5.3. Label-Free Feature Selection

Three feature-ranking criteria that do not use the labels were compared with the BCV ranking on the 85 features of Method 5 (Table 9): the Laplacian score [29], the variance-weighted PCA loading of each feature on the first five principal components, and a greedy correlation-redundancy filter that removes features correlated at |r| > 0.9 with a retained feature. Clustering with all 85 features and with 200 random subsets of 5, 10 and 15 features served as reference points. None of the label-free criteria recovered the class structure: the ARI values lay within ±0.1 of zero for all subset sizes (the only exception being the correlation filter with five features, 0.18), as did clustering with all features and with random subsets, whereas the BCV ranking gave 0.58–0.62 with 5–10 features. The same result was obtained when the label-free rankers were used inside the LOSO loop. The class structure recovered by the framework therefore resides in a small, label-informed subset of the features and is not the dominant source of variance in the feature space. For this reason, the framework is described as label-informed feature ranking followed by unsupervised clustering rather than as an unsupervised method, and the nested estimates are regarded as its fair performance figures.

#### 4.5.4. Contribution of the Detector Features (Ablation)

The contribution of the three detector features of Method 5 that entered the top 15 (of the 25 features added in Method 5) was tested by a controlled comparison in which only the candidate feature set differed (Method 4 versus Method 5) while the number of selected features, the seeds, and the selection criterion were held fixed (Table 10). With five features, the two methods selected the same features on the full data and were identical; in one of the 43 LOSO folds, Method 5 selected sse_num_events instead of sp_propagation_extent, which changed a single prediction (nested LOSO ARI 0.402 vs. 0.439). With ten features, Method 5 was higher in the full-data evaluation (ARI 0.621 vs. 0.567; paired difference +0.054, 95% CI 0.051 to 0.059) but lower under nested LOSO (0.419 vs. 0.537). With fifteen features, Method 4 collapsed to 0.22 while Method 5 remained at 0.58, but both were below 0.3 under nested validation. Within the Method 5 top-15 set, removing the three detector features changed the seed-averaged ARI from 0.579 to 0.550. The detector features therefore carry complementary information but do not improve the classification consistently, and they are not part of the recommended configuration.

#### 4.5.5. Class-Specific Performance

Because missing a genuine displacement is operationally far more serious than misclassifying a malfunction, Table 11 reports precision and recall per class, the number of missed Actual Displacement events and the corresponding false-negative rate (FNR) for the recommended configuration, and Figure 10 shows the confusion matrices under nested LOSO validation. The precision of the Actual Displacement class was 1.0 in every evaluation: no Malfunction or Others event was ever assigned to the displacement cluster. Its recall, however, was 0.615 with the 8 h window (8 of 13 events; FNR 0.38, 95% Wilson CI 0.18–0.64). The five missed events all occurred at densely instrumented stations (69–112 displacement sensors) and had network-wide synchronization ratios between 0.24 and 0.50, i.e., the displacement was recorded by only part of a large sensor network. Malfunction was recovered with recall 0.93 and Others with recall 0.67 under LOSO; the Others errors are confusions with Malfunction, consistent with the two-stage structure of the classification rules (Table 7). With the alarm-time window, the recall of Actual Displacement rose to 0.923 (12 of 13; FNR 0.08, 0.01–0.33) while precision remained 1.0.

Because the recall of Actual Displacement was the primary selection criterion, the recommended configuration was also compared with alternatives designed specifically to maximize it (Table 12). Ranking the features by their ability to separate Actual Displacement from the two other classes (BCV ratio computed for Actual Displacement against all other events) selected the same three synchronization features first, but the features added in fourth and fifth place were trend features that lowered the recall of Actual Displacement to 0.54 in both windows. A dedicated two-cluster stage that separates Actual Displacement from all other events using only the three synchronization features recovered exactly the same events as the three-cluster solution (8 of 13 with the 8 h window and 12 of 13 at the alarm time). Among the 30 method/feature-number combinations, the ten-feature configurations of Methods 3–5 recovered one more event with the 8 h window (9 of 13) at the cost of one false displacement assignment and of seed dependence, and fell to 7 of 13 at the alarm time. The events missed with the 8 h window are therefore not recovered by any selection strategy: they are determined by the analysis window and by the network-wide definition of the synchronization features, not by the selection criterion.

### 4.6. Evaluation with Data Available at the Alarm Time

**Figure 16:**
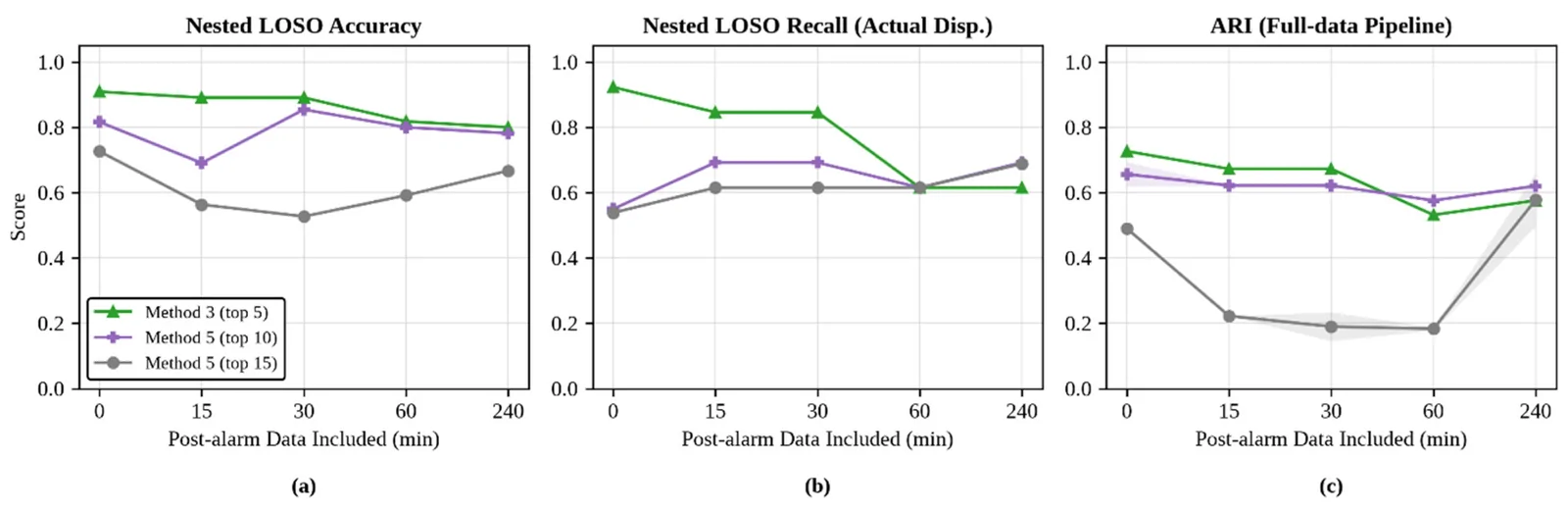
Effect of the post-alarm data included in the analysis window on (**a**) nested LOSO accuracy, (**b**) nested LOSO recall of Actual Displacement, and (**c**) the seed-averaged ARI of the full-data pipeline, for three configurations. LOSO, leave-one-site-out; ARI, adjusted Rand index.

All results so far use a window that extends 4 h beyond the alarm time t0, so they could not be reproduced by an operational system at the moment the alarm is issued. To assess this, the features were recomputed from the data available at t0 ([t0 − 4 h, t0]) and after 15, 30, and 60 min of post-alarm data, and the complete validation procedure—seed-averaged full-data evaluation, BCV ranking inside the LOSO loop, bootstrap confidence intervals, and permutation test—was repeated for each window (Table 13, Figure 16).

Restricting the analysis to the alarm time did not degrade the classification; on the contrary, the point estimates improved. With the five-feature configuration the seed-averaged ARI of the full-data pipeline rose from 0.576 (8 h window) to 0.727 at t0, and under nested LOSO the accuracy rose from 0.800 to 0.909 (95% CI 0.84–0.98), balanced accuracy to 0.897 (0.80–0.98), macro-F1 to 0.904, Cohen’s κ to 0.847 and ARI to 0.727 (0.53–0.94); 12 of the 13 Actual Displacement events, 28 of 30 Malfunction events and 10 of 12 Others events were recovered (Figure 10b), the silhouette score rose from 0.452 to 0.564, and the permutation test again gave *p* = 0.001. Of the 55 events, 7 were classified correctly at t0 but not with the 8 h window and 1 the other way round (McNemar exact test *p* = 0.07); the confidence intervals of the two accuracies overlap, so the improvement is suggestive rather than conclusive at this sample size. The full-data and nested estimates coincided at t0, indicating that the feature ranking was identical in every LOSO fold. Performance then decreased as post-alarm data were added (LOSO accuracy 0.891 at +15 min and +30 min, 0.818 at +60 min and 0.800 at +4 h). The configurations that rely on the detector and trend features of Method 5 behaved differently: Method 5 with 10 features was competitive at t0 (LOSO accuracy 0.816) but unstable at intermediate windows, and Method 5 with 15 features performed poorly at every window shorter than 4 h.

Two control analyses confirm that the gain is due to the pre-alarm content of the window and not to its shorter length or to the change of the fifth feature (Table 13, last rows). A window of the same 4 h duration placed entirely after the alarm ([t0, t0 + 4 h]) was almost uninformative under nested validation (LOSO accuracy 0.547—equal to the majority-class rate of 0.545—LOSO ARI 0.03, recall of Actual Displacement 0.32; the full-data pipeline reached an ARI of only 0.32 and selected none of the synchronization-lag features: sp_xy_tilt_corr, ts_max_cross_corr, ts_propagation_direction, ts_waveform_rise_time and sp_colocated_corr_avg), and a 4 h window centered on the alarm ([t0 − 2 h, t0 + 2 h]) was intermediate (LOSO accuracy 0.855, ARI 0.627). Applying the five features selected with the 8 h window to the alarm-time data gave a LOSO accuracy of 0.891 (ARI 0.681), and applying the alarm-time features to the 8 h data gave 0.818 (0.490); the window, not the feature set, drives the difference. The reference time t0 is the alarm time stored with each exported record by the monitoring system, which issues an alarm when a displacement of 10 mm or a tilt of 1° is exceeded; a cross-check against the raw records showed that for 53 of the 55 events a labeled sensor first exceeded this threshold within 30 min of t0 (median 1.9 min before t0; 44 events within 10 min), consistent with the 1 min sampling interval and the processing delay of the system. The pre-alarm window therefore ends at the moment an operator would receive the alarm.

Two mechanisms explain the improvement. First, the alarm is triggered when the displacement threshold is exceeded, so the pre-alarm window contains the onset of the event, where the distinguishing patterns are cleanest: neighboring sensors respond within a few minutes of each other in a genuine displacement, whereas a malfunction produces an isolated or irregular change. Second, the post-alarm period adds signal components that blur these patterns—displaced sensors settling into a new equilibrium or saturating, malfunctioning sensors producing further irregular values or being reset, and field-response activities—and these components shift the cross-correlation maxima of several Actual Displacement events to long lags. For three of the five events missed with the 8 h window, ts_max_lag increased from 1–4 min at t0 to 4–10 min when the post-alarm data were included, which moved them into the asynchronous region of the feature space. Consistently, the BCV importance of the synchronization and co-located correlation features was higher at t0 than with the 8 h window (ts_sync_ratio 0.71 vs. 0.60; sp_colocated_corr_avg 0.49 vs. 0.19), whereas the trend and step-change features of Methods 4–5, which require a longer record, lost most of their discriminative power (tr_avg_confidence 0.06 vs. 0.18). At t0, the fifth selected feature was sp_colocated_corr_avg instead of sp_propagation_extent; the three synchronization features and sp_xy_tilt_corr were selected in every window.

These results address the question of real-time applicability: the cause classification can be issued together with the alarm itself, using the 4 h of data preceding it, and does not require post-alarm data. Two caveats apply. One Malfunction event had only 17 min of pre-alarm data in its record, so its alarm-time features were computed from a short segment; and, as for all results of this study, the confidence intervals are wide because only 13 Actual Displacement events are available.

### 4.7. Number of Clusters

**Figure 17:**
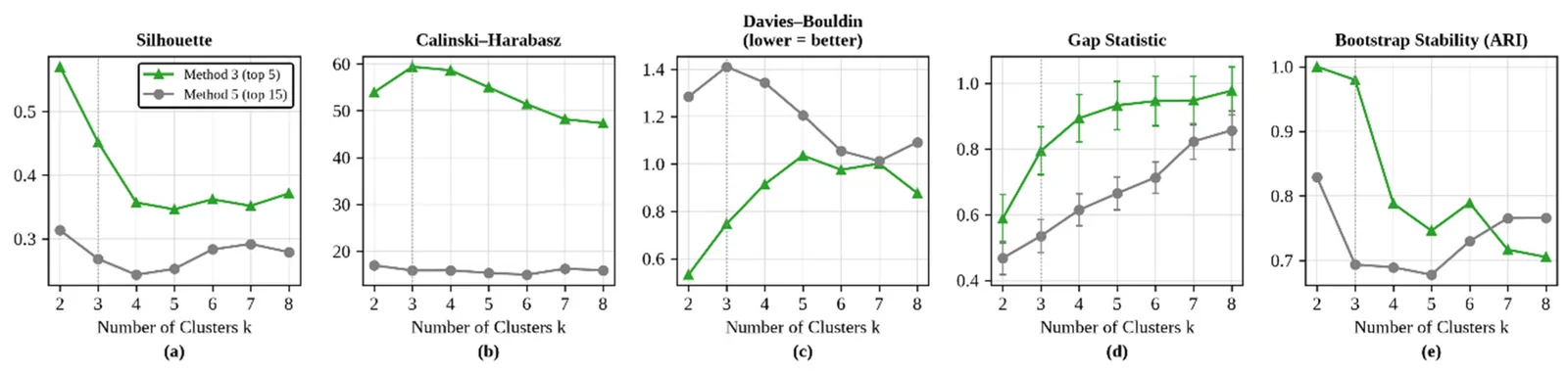
Cluster-number criteria for k = 2–8 for the recommended five-feature set and the 15-feature set of Method 5: (**a**) silhouette, (**b**) Calinski–Harabasz, (**c**) Davies–Bouldin, (**d**) gap statistic (bars: ± one standard error) and (**e**) bootstrap stability. The dotted line marks k = 3. ARI, adjusted Rand index.

The number of clusters k = 3 was imposed to match the three operational alarm categories. To examine whether three groups are also supported by the intrinsic structure of the data, k = 2–8 were compared for the recommended five-feature set and for the 15-feature set of Method 5 using the silhouette score, the Calinski–Harabasz and Davies–Bouldin indices, the gap statistic [30] (100 uniform reference samples) and a bootstrap stability criterion (mean ARI between k-means solutions fitted on two random 80% subsamples, 100 pairs) (Figure 17). For the five-feature set, the silhouette (0.57 vs. 0.45), Davies–Bouldin, and stability criteria preferred k = 2, the Calinski–Harabasz index preferred k = 3, and the gap criterion selected k = 4; for the 15-feature set, every criterion except the gap statistic preferred k = 2. The k = 2 solution separates the eight highly synchronized Actual Displacement events from all other events, whereas k = 3 additionally isolates a cluster dominated by Others ([[8, 0, 0], [5, 29, 2], [0, 1, 10]], listed as [AD, MF, OT] counts per cluster). The signal structure therefore supports a strong two-way distinction between synchronous displacement and asynchronous non-displacement events, and a weaker third group; k = 3 is retained because it corresponds to the three operational response categories and is supported by the Calinski–Harabasz index, but the weaker intrinsic support for the third cluster is consistent with the two-stage form of the classification rules (Table 7), in which the Others–Malfunction distinction relies on a single spatial-correlation feature.

This two-way distinction is also the part of the framework that does not require the labels. If the three synchronization features are fixed in advance rather than selected by the BCV ranking—their role follows from the physical premise of the framework (Section 1 and Section 2.3)—and the displacement cluster is identified by a rule rather than by Hungarian matching (the cluster with the higher mean ts_sync_ratio), no label enters the analysis at any step. This label-free two-cluster detector classifies 50 of the 55 events correctly with the 8 h window and 54 of 55 with alarm-time data (accuracy 0.909 and 0.982, recall of Actual Displacement 8 of 13 and 12 of 13, with no non-displacement event assigned to the displacement cluster), reproducing the two-cluster row of Table 12; because nothing is fitted to the labels, its nested leave-one-site-out estimates coincide with the full-data values. The label-informed ranking is therefore necessary for the three-class problem—the same three features with k = 3 reach an ARI of only 0.274, and no label-free ranking criterion recovers the three-class structure (Section 4.5.3)—but not for the displacement/non-displacement separation.

### 4.8. Sensitivity to Missing Data and Sensor Count

Missing observations in the raw records were rare (Table 14): the mean fraction of missing cells among the displacement-sensor columns was 1.78% for Actual Displacement, 0.51% for Malfunction and 0.00% for Others, with a maximum of 22.9% in a single soil-loss event, and nine records were shorter than the nominal 480 rows (minimum 257 rows), owing either to a late start of the record (one Malfunction event whose record begins 17 min before the alarm) or to internal gaps. Because the extractors compute each feature on the available observations, no missing or infinite values occurred in the final feature matrix, and replacing the raw gaps with the column median or excluding the affected features instead gave results identical to those reported.

**Figure 18:**
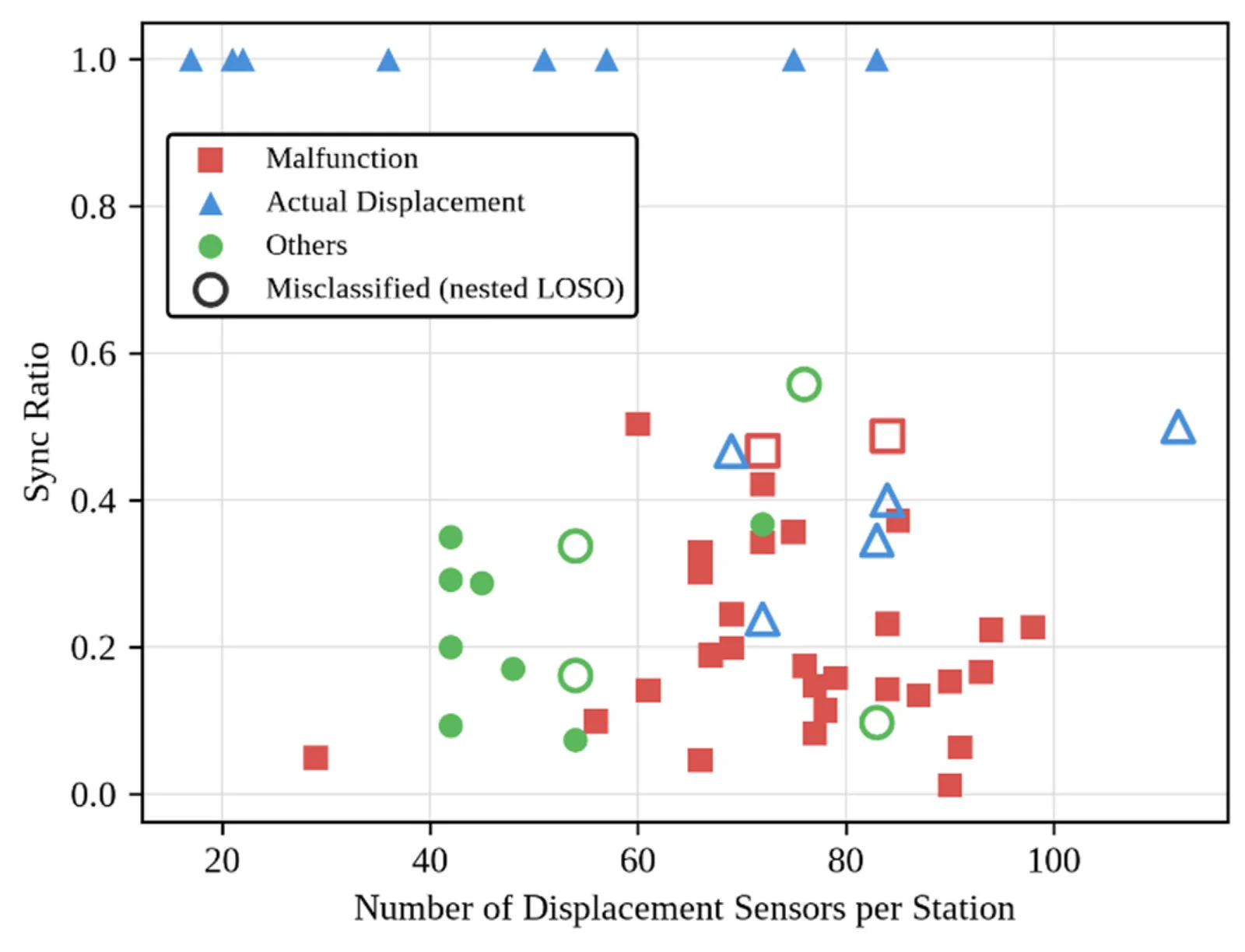
Network-wide synchronization ratio (ts_sync_ratio) against the number of displacement sensors per station, by field label; open symbols mark events misclassified under nested LOSO (8 h window, recommended configuration). Squares, triangles, and circles indicate Malfunction, Actual Displacement, and Others events, respectively, and filled symbols mark correctly classified events. LOSO, leave-one-site-out.

The stations differ considerably in size (17–112 displacement sensors). Under nested LOSO with the 8 h window, the proportion of correctly classified events decreased with the number of sensors (accuracy 0.89, 0.78, and 0.72 in the lower, middle, and upper tertiles of sensor count; point-biserial correlation r = −0.23, *p* ≈ 0.10, i.e., not significant at n = 55), and all five missed Actual Displacement events belong to stations with at least 69 sensors, whereas every Actual Displacement event in the lower tertile was recovered (Figure 18). The synchronization ratio is computed over all sensor pairs of a station, so a displacement that affects a limited part of a large network yields a low network-wide ratio and is confused with a malfunction. Computing the synchronization features within the neighborhood of the alarming sensor, or normalizing them by the number of responding sensors, is therefore the most promising direction for improving recall at large stations; with the alarm-time window, this effect was already reduced to a single missed event. Sensor coordinates were not available, so the sensitivity to sensor spacing could not be evaluated.

## 5. Discussion

### 5.1. Interpretation of Results

The principal finding of this study is a complementary structure in which time-series synchronization features play a decisive role in identifying Actual Displacement events, whereas spatial correlation features enable fine-grained differentiation among non-displacement events. Owing to this structural characteristic, applying individual feature groups independently is insufficient to classify all three cause categories simultaneously. An integrated application is therefore essential. The detector-based features newly introduced in Method 5 were distributed within importance ranks 10 to 15, but the controlled ablation showed that they do not consistently improve the agreement with the field labels once seed dependence and label leakage are controlled; the recommended configuration therefore uses only synchronization and spatial-correlation features.

Under the recommended configuration, the silhouette score (0.452 with the 8 h window, 0.564 with the alarm-time window) indicates moderate rather than strong intrinsic separation: the highly synchronized Actual Displacement events form a compact and well-separated group, whereas the remaining Actual Displacement events and the Malfunction and Others events overlap in feature space, and the agreement with the field labels in this region is obtained by centroid-based partitioning. The external metrics should therefore be read together with their confidence intervals and with the class-specific recall, rather than as evidence of clearly separated groups; the nested leave-one-site-out estimates are the fair figures, and the full-data values are upper bounds. Purity is also sensitive to the class distribution (Malfunction accounts for 54.5% of the events), which is why balanced accuracy, macro-F1 and Cohen’s κ are reported alongside it. Finally, the larger 15-feature configuration had partly learned station-specific structure of the Others events, which becomes visible only under site-wise validation; this observation underlines the importance of site-wise validation when the events are concentrated at a few stations.

### 5.2. Comparison with Previous Studies

Previous studies [12,13,14,15,31] on slope monitoring have primarily relied on threshold-based alarm systems derived from absolute sensor values or rates of change. Persistent false alarm issues have arisen due to the inability to distinguish sensor malfunction signals from actual ground displacement signals. In contrast, this study does not depend on absolute values from individual sensors but proposes a novel approach that classifies displacement causes based on relative multi-sensor patterns, including time lags, spatial correlations, and signal synchronization characteristics.

Moreover, while most research in industrial IoT anomaly detection focuses on binary classification between normal and abnormal states, this study classifies anomalies into three categories: Malfunction, Actual Displacement, and Others. In the proposed framework, the expert labels are used only to rank the candidate features, and the partitioning of events is performed by unsupervised clustering; the results show that a compact set of label-informed, physically interpretable features allows the three cause categories to be recovered to a substantial degree by the clustering step, with a leave-one-site-out accuracy comparable to that of supervised classifiers trained on the same features. The resulting framework provides interpretability and practical applicability.

### 5.3. Practical Implementation

The classification rules listed in Table 7 can be incorporated into a multi-level alarm-handling procedure once their thresholds have been re-derived from alarm-time data. Because the classification obtained from the data recorded up to the alarm time was at least as good as with any post-alarm window, the cause classification can be issued together with the alarm itself, from the preceding 4 h of data, and does not need to wait for post-alarm data; the framework is nevertheless not a replacement for the threshold-based alarm, which detects the event, but a decision-support module that classifies its probable cause. Within this procedure, events satisfying the time-series synchronization conditions may be escalated as probable displacement for immediate field inspection, whereas among asynchronous events the spatial-correlation characteristics can guide recommendations for sensor inspection or maintenance. Because the five selected features are inexpensive to compute, the module can be integrated into existing threshold-based alarm systems, and the identification of Malfunction events can enhance alarm reliability and support the prioritization of sensor maintenance.

For a safety-related application, the cost of missing an Actual Displacement event is far higher than that of an unnecessary inspection. The decision boundary for the Actual Displacement category should therefore be set conservatively, accepting additional false positives in order to minimize false negatives, and the class-specific recall and false-negative rate reported in Table 11 provide the basis for this setting: in the present dataset, no non-displacement event was ever assigned to the displacement cluster, whereas 1 of 13 (alarm-time window) to 5 of 13 (8 h window) displacement events were missed, all at densely instrumented stations. At the current stage, the framework should be regarded as a decision-support filter operating alongside the existing threshold alarm, not as a stand-alone criterion for suppressing alarms.

### 5.4. Limitations

This study is an exploratory investigation based on empirical data collected from an operational IoT-based slope monitoring system and has several limitations.

First, the dataset consists of 55 event samples, of which only 13 are Actual Displacement events. Although the events were collected from 43 monitoring stations, this sample size—small relative to the 85 candidate features—limits statistical generalization, and the reported performance should be regarded as exploratory. Second, no independent external dataset was available for validation. The leave-one-site-out and resampling analyses are the closest available substitute, but validation on newly accumulated events remains necessary, and the confidence intervals reported here are wide because only 13 Actual Displacement events are available. Third, the Others events are concentrated at a small number of stations (seven of the twelve events at a single station), so the characteristics attributed to this class may partly reflect site-specific conditions. Fourth, the spatial correlation and time-series synchronization features depend on the sensor deployment, in particular the number and spacing of sensors. The stations analyzed here differ considerably in sensor count (17–112 sensors), and all displacement events missed with the 8 h window were found at stations with at least 69 sensors; sensor coordinates were not available, however, so sensitivity to sensor spacing could not be assessed. When the framework is transferred to a new site, we suggest the following recalibration procedure: (i) derive site-specific reference distributions of the synchronization features (e.g., ts_sync_ratio and ts_max_lag) from an initial period of routine operation; (ii) confirm the cluster-to-cause mapping using the first expert-verified alarms at the site; and (iii) re-derive the reference distributions whenever sensors are replaced or added. Fifth, rainfall, although recorded at every station, was not included in the analysis because the number of events accumulated so far is too small to evaluate its contribution reliably, and the monitoring network does not include suction, pore-water pressure, or soil-moisture sensors; integrating rainfall and such hydro-mechanical measurements with the displacement records is a natural extension towards a more physically informed early-warning framework. Finally, practical implementation may benefit from a semi-supervised approach that incorporates the limited labeled data.

## 6. Conclusions

This study devised a clustering-based methodology to automatically classify displacement alarm causes using sensor monitoring data collected from an IoT-based slope monitoring system; the methodology was evaluated using real operational data. Time-series data from a multi-sensor network consisting of inclinometers and extensometers were used to extract 85 domain-based features. Feature selection and clustering were applied to classify three cause categories: Malfunction, Actual Displacement, and Others. The main conclusions are as follows.

(1) Through systematic comparison of five feature extraction methods and feature subset sizes, evaluated over 100 random seeds, a compact configuration of five features (three cross-correlation-based synchronization features and two spatial-correlation features) was identified that is stable across seeds and identical for Methods 3–5 on the full data. Under nested leave-one-site-out validation, which removes the label information shared between feature ranking and evaluation, it classified 80% of the events correctly (95% CI 69–89%; ARI 0.44) with the full 8 h window and 91% (84–98%; ARI 0.73) when only data available at the alarm time were used. Because label-free feature selection did not recover the class structure, the framework is characterized as label-informed feature ranking followed by unsupervised clustering.
(2) Cross-correlation-based time-series synchronization features (ts_max_lag, ts_avg_lag, and ts_sync_ratio) exhibited the highest discriminative power, confirming that inter-sensor time-lag patterns are a primary determinant in displacement cause classification. Actual ground displacement events showed rapid synchronization responses within an average of 1.05 min and a mean synchronization ratio of 0.77, whereas Malfunction events exhibited a maximum lag of 10 min and a mean synchronization ratio of 0.22. This demonstrates the physical distinction between displacement signal propagation and sensor noise; displacement events that involved only part of a large sensor network were, however, not recovered with the 8 h window.
(3) The spatial correlation feature sp_xy_tilt_corr enabled fine-grained classification between asynchronous events, specifically Malfunction and Others, which are difficult to distinguish using time-series features alone. This indicates that complementary integration of temporal and spatial features is critical for classifying all three categories, although the intrinsic support for the third cluster is weaker than for the displacement/non-displacement distinction.
(4) The three features derived from the sudden-synchronization and malfunction-pattern detectors entered the top 15 of the label-informed ranking but did not improve the classification consistently in a controlled ablation; the recommended configuration therefore consists of the five synchronization and spatial-correlation features only.
(5) By analyzing the physical meaning of key features, interpretable classification rules understandable to field engineers were derived. These rules provide a practical foundation for multi-level decision criteria in alarm-handling systems, and the evaluation with alarm-time data shows that the classification can be issued together with the alarm itself; because no non-displacement event was assigned to the displacement cluster while some displacement events were missed, the displacement boundary should be set conservatively in operational use.

The most significant contribution of this study is moving beyond conventional absolute-value-based threshold alarm approaches and proposing a displacement cause classification framework grounded in relative temporal and spatial multi-sensor patterns. Classifying about four out of five alarms correctly under site-wise validation—and about nine out of ten from alarm-time data—with only five interpretable features demonstrates that the approach is computationally light and interpretable, and supports its use as a decision-support filter alongside threshold alarms in operational slope-monitoring systems, subject to validation on larger datasets.

This study is exploratory in nature and has limitations, including the limited sample size, the absence of external validation, the site concentration of the Others events, and site dependency, as discussed above. Future research should focus on validation using newly accumulated events from diverse sites, integration of rainfall and hydro-mechanical measurements such as soil suction and pore-water pressure, performance comparison with supervised classifiers, hybrid approaches integrating time-series deep learning models with domain-based features, and pilot implementation in real-time monitoring systems to further extend generalizability and practical applicability of the proposed framework. In addition, future research should evaluate and extend the applicability of the proposed framework across various slope environments, including reservoir embankments, natural slopes surrounding dams, embankment (fill) slopes, cut slopes, and roadside slopes.

## Figures and Tables

**Table 1:** Configuring datasets by event type.

Event	Label	Samples	Ratio
Malfunction	100	30	54.5%
Actual Displacement	300	13	23.6%
Others	400	12	21.8%
Total	-	55	100%

**Table 2:** Comparison of the number of features by feature extraction method.

Method	Description	Feature Group	Number of Features
Method 1	Statistical + Waveform	Statistics, spikes, waveforms, cross-correlation	33
Method 2	Spatial + Trend	Spatial correlation, trends	21
Method 3	Hybrid + Trend	Method 1 + Method 2 integration	54
Method 4	Hybrid + Trend + Step	Method 3 + Step Change Detection	60
Method 5	All Features	Method 4 + Rapid Synchronization + Malfunction Pattern + Event-classifier indicators (signal-derived)	85

**Table 3:** Top 15 core features (based on Method 5).

Rank	Feature Name	Importance	Category	Physical Meaning
1	ts_max_lag	0.664	Time-series synchronization	Maximum inter-sensor time delay
2	ts_avg_lag	0.639	Time-series synchronization	Average inter-sensor time delay
3	ts_sync_ratio	0.595	Time-series synchronization	Synchronization event ratio
4	sp_xy_tilt_corr	0.457	Spatial correlation	X–Y axis slope correlation coefficient
5	sp_propagation_extent	0.251	Spatial correlation	Event propagation range
6	ts_inter_event_std	0.221	Time series timing	Inter-event interval standard deviation
7	sp_colocated_corr_avg	0.194	Spatial correlation	Average same-location sensor correlation
8	ts_inter_event_mean	0.177	Time series timing	Inter-event interval average
9	tr_avg_confidence	0.176	Trend	Average trend confidence
10	sse_num_events ^1^	0.175	Rapid synchronization	Number of sudden synchronization events
11	num_multi_sensor_events	0.167	Event characteristics	Number of simultaneous multi-sensor events
12	sc_avg_confidence	0.161	Step change	Average confidence of detected step changes
13	mf_inclinometer_malfunction_ratio ^1^	0.155	Malfunction pattern	Inclinometer malfunction ratio
14	tr_avg_r_squared	0.152	Trend	Trend determination coefficient
15	sse_event_coverage ^1^	0.151	Rapid synchronization	Rapid synchronization coverage

^1^ Indicates newly introduced detector features in Method 5.

**Table 4:** Clustering performance by number of features (k-means; ARI given as mean ± SD over 100 random seeds).

Method	Number of Features	Algorithm	ARI	NMI	Silhouette Score	Purity
Method 1	5	k-means	0.221 ± 0.000	0.351	0.392	70.9%
Method 1	10	k-means	0.216 ± 0.007	0.352	0.419	70.9%
Method 1	15	k-means	0.224 ± 0.000	0.349	0.370	70.9%
Method 2	5	k-means	0.347 ± 0.000	0.276	0.432	70.9%
Method 2	10	k-means	0.357 ± 0.012	0.276	0.298	70.7%
Method 2	15	k-means	0.014 ± 0.005	0.103	0.238	56.4%
Method 3	5	k-means	0.576 ± 0.000	0.578	0.452	85.5%
Method 3	10	k-means	0.567 ± 0.023	0.565	0.315	85.3%
Method 3	15	k-means	0.224 ± 0.000	0.349	0.278	70.9%
Method 4	5	k-means	0.576 ± 0.000	0.578	0.452	85.5%
Method 4	10	k-means	0.567 ± 0.023	0.565	0.315	85.3%
Method 4	15	k-means	0.221 ± 0.015	0.375	0.223	70.9%
Method 5	5	k-means	0.576 ± 0.000	0.578	0.452	85.5%
Method 5	10	k-means	0.621 ± 0.000	0.609	0.339	87.3%
Method 5	15	k-means	0.579 ± 0.082	0.571	0.266	85.5%

ARI, adjusted Rand index; NMI, normalized mutual information; SD, standard deviation.

**Table 5:** Performance comparison by clustering algorithm (Method 3, top 5; 8 h window).

Algorithm	Number of Clusters	ARI	NMI	Silhouette Score	Purity
k-means	3	0.576	0.578	0.452	85.5%
Hierarchical	3	0.525	0.534	0.451	83.6%
DBSCAN	2	0.259	0.363	0.570	69.1%

ARI, adjusted Rand index; NMI, normalized mutual information; DBSCAN, density-based spatial clustering of applications with noise.

**Table 6:** Statistics by event type for key features (mean ± standard deviation).

Feature Name	Malfunction (100)	Actual Displacement (300)	Others (400)	Significance
ts_max_lag	**10.00 ± 0.00**	3.15 ± 4.26	**10.00 ± 0.00**	Actual displacement (300): less than 10 min
ts_avg_lag	5.95 ± 1.57	**1.05 ± 1.64**	5.86 ± 1.42	Fast response only for actual displacement (300)
ts_sync_ratio	0.22 ± 0.13	**0.77 ± 0.30**	0.25 ± 0.14	High synchronization only for actual displacement (300)
sp_xy_tilt_corr	0.01 ± 0.23	0.10 ± 0.16	**0.54 ± 0.27**	High correlation only for others (400)
sp_propagation_extent	**5.92 ± 1.89**	3.76 ± 2.00	3.73 ± 1.66	Widest range of failures

Numbers in parentheses are the label codes of the event types (100, Malfunction; 300, Actual Displacement; 400, Others). Values in bold indicate the event type(s) for which each feature takes a characteristic value that distinguishes them from the other types.

**Table 7:** Feature-based event classification rules.

Condition	Classification	Physical Basis
ts_sync_ratio > 0.5 AND ts_avg_lag < 2 min	Actual Displacement (300)	High sync. + fast response = physical wave propagation
ts_max_lag = 10 min AND sp_xy_tilt_corr > 0.3	Others (400)	Asynchronous + consistent direction = external physical disturbance
ts_max_lag = 10 min AND sp_xy_tilt_corr ≤ 0.3	Malfunction (100)	Asynchronous + uncorrelated = sensor noise

**Table 8:** Performance under different validation schemes (8 h window; nested k-means estimates are means over 20 seeds with 95% bootstrap confidence intervals; the supervised baselines are point estimates from the same nested LOSO procedure using the same five features).

Configuration	Scheme	Accuracy	Balanced Accuracy	Macro-F1	ARI
Method 3, top 5	Full data	0.855	0.805	0.837	0.576
	Nested LOEO	0.84 (0.73–0.93)	0.79 (0.66–0.90)	0.82 (0.69–0.92)	0.53 (0.32–0.77)
	Nested LOSO	0.80 (0.69–0.89)	0.74 (0.59–0.87)	0.77 (0.62–0.88)	0.44 (0.23–0.68)
Method 5, top 10	Full data	0.873	0.831	0.860	0.621
	Nested LOEO	0.78 (0.67–0.89)	0.73 (0.59–0.85)	0.75 (0.60–0.86)	0.43 (0.21–0.67)
	Nested LOSO	0.78 (0.62–0.89)	0.73 (0.51–0.88)	0.75 (0.51–0.88)	0.42 (0.17–0.68)
Method 5, top 15	Full data	0.852	0.818	0.840	0.579
	Nested LOEO	0.82 (0.69–0.93)	0.77 (0.62–0.90)	0.80 (0.65–0.91)	0.50 (0.25–0.77)
	Nested LOSO	0.67 (0.51–0.82)	0.58 (0.44–0.74)	0.59 (0.45–0.75)	0.28 (0.11–0.53)
Supervised baselines	Logistic regression	0.800	0.715	0.743	0.480
	Linear discriminant analysis	0.764	0.664	0.702	0.375

LOEO, leave-one-event-out; LOSO, leave-one-site-out; ARI, adjusted Rand index; Macro-F1, unweighted mean of the class-wise F1 scores.

**Table 9:** Clustering performance (k-means, ARI mean ± SD over 100 seeds) with label-informed and label-free feature selection on the 85 features of Method 5 (8 h window).

Ranking Criterion	Top 5	Top 10	Top 15
BCV (label-informed)	0.576 ± 0.000	0.621 ± 0.000	0.579 ± 0.082
Laplacian score	−0.072 ± 0.015	0.010 ± 0.000	0.030 ± 0.004
PCA loading	−0.033 ± 0.000	−0.033 ± 0.000	−0.031 ± 0.000
Correlation filter	0.178 ± 0.004	0.051 ± 0.015	0.080 ± 0.002
Random subsets (200 draws)	0.026 ± 0.087	0.032 ± 0.086	0.011 ± 0.072
No selection (all 85 features)	−0.029 ± 0.017		

BCV, between-class variance; PCA, principal component analysis; ARI, adjusted Rand index; SD, standard deviation.

**Table 10:** Clean ablation of the detector features: Method 4 versus Method 5 at equal numbers of selected features (8 h window; ARI mean over 100 seeds; the CI of the difference is a paired bootstrap over seeds and reflects initialization variability only, not sampling uncertainty; nested LOSO ARI mean over 20 seeds).

Top-N	ARI Method 4	ARI Method 5	Difference (95% CI)	Nested LOSO M4	Nested LOSO M5	Detector Features in M5 top-N
5	0.576	0.576	+0.000 (+0.000, +0.000)	0.439	0.402	0
10	0.567	0.621	+0.054 (+0.051, +0.059)	0.537	0.419	1
15	0.221	0.579	+0.357 (+0.339, +0.373)	0.134	0.284	3
20	0.221	0.216	−0.005 (−0.008, −0.002)	0.294	0.242	4

N, number of selected features; ARI, adjusted Rand index; CI, confidence interval; LOSO, leave-one-site-out; M4, Method 4; M5, Method 5.

**Table 11:** Class-specific performance of the recommended configuration (Method 3, top 5, k-means). Nested values are means over 20 seeds. AD = Actual Displacement, MF = Malfunction, OT = Others.

Evaluation	Recall AD	Recall MF	Recall OT	Precision AD	Precision MF	Precision OT	Missed AD (FNR)
8 h window, full data	0.615	0.967	0.833	1.000	0.806	0.909	5 of 13 (0.38)
8 h window, nested LOSO	0.615	0.933	0.667	1.000	0.757	0.800	5 of 13 (0.38)
Alarm-time window, nested LOSO	0.923	0.933	0.833	1.000	0.903	0.833	1 of 13 (0.08)

FNR, false-negative rate; LOSO, leave-one-site-out.

**Table 12:** Recall of Actual Displacement under nested leave-one-site-out validation for the recommended configuration and for alternative selection strategies aimed at the Actual Displacement class (Method 3 candidate set; k-means, means over 20 seeds; AD = Actual Displacement, OT = Others).

Selection Strategy	Window	Recall AD (Missed of 13)	Precision AD	Recall OT	Accuracy
Three-class BCV ranking, top 5 (recommended)	8 h	0.615 (5)	1.000	0.667	0.800
	Alarm time	0.923 (1)	1.000	0.833	0.909
Three-class BCV ranking, top 10	8 h	0.692 (4)	0.975	0.667	0.836
	Alarm time	0.538 (6)	1.000	0.738	0.815
AD-vs-rest BCV ranking, top 5	8 h	0.538 (6)	1.000	0.417	0.709
	Alarm time	0.538 (6)	1.000	0.000	0.655
AD-vs-rest BCV ranking, top 10	8 h	0.612 (5.1)	1.000	0.167	0.654
	Alarm time	0.615 (5)	0.989	0.462	0.499
Two-cluster stage (k = 2) ^2^	8 h	0.615 (5)	1.000	–	0.909 ^1^
	Alarm time	0.923 (1)	1.000	–	0.982 ^1^

^1^ Accuracy of the two-class assignment (Actual Displacement versus all other events). ^2^ Two clusters fitted on the three synchronization features only. BCV, between-class variance.

**Table 13:** Performance of the recommended configuration as a function of the analysis window (ARI and purity: full-data pipeline, mean over 100 seeds; LOSO columns: nested leave-one-site-out, mean over 20 seeds). The last two rows are window-length controls of the same 4 h duration as the alarm-time window.

Analysis Window	ARI (Full Data)	Purity (Full Data)	LOSO Accuracy	LOSO Balanced Accuracy	LOSO Recall AD	Missed AD	Fifth Selected Feature
0 min (alarm time)	0.727	90.9%	0.909	0.897	0.923	1 of 13	sp_colocated_corr_avg
15 min	0.673	89.1%	0.891	0.871	0.846	2 of 13	sp_colocated_corr_avg
30 min	0.673	89.1%	0.891	0.871	0.846	2 of 13	sp_colocated_corr_avg
60 min	0.532	83.6%	0.818	0.766	0.615	5 of 13	sp_colocated_corr_avg
240 min (8 h window)	0.576	85.5%	0.800	0.738	0.615	5 of 13	sp_propagation_extent
Control: 4 h after alarm only [t0, t0 + 4 h]	0.322	74.0%	0.547	0.427	0.319	8.8 of 13 (seed mean)	none of the lag features (see text)
Control: 4 h centered on alarm [t0 − 2 h, t0 + 2 h]	0.627	87.3%	0.855	0.849	0.846	2 of 13	sp_colocated_corr_avg

ARI, adjusted Rand index; LOSO, leave-one-site-out; AD, Actual Displacement.

**Table 14:** Missing data in the raw event records by class (displacement-sensor columns; 480 rows nominal).

Class	Events	Mean Missing Cells	Max. Missing Cells	Mean Row Deficit	Max. Row Deficit
Actual Displacement	13	1.78%	22.9%	10.8 rows	125 rows
Malfunction	30	0.51%	11.3%	18.9 rows	223 rows
Others	12	0.00%	0.0%	7.4 rows	59 rows

## Data Availability

The data that support the findings of this study were obtained through a commissioned project funded by the Chungju National Land Management Office, Ministry of Land, Infrastructure and Transport (MOLIT), Republic of Korea (Project No. 20250684), and are not publicly available due to institutional restrictions. The data may be available from the corresponding author upon reasonable request and with permission from the Chungju National Land Management Office.

## Abbreviations

The following abbreviations are used in this manuscript:

IoT	Internet of Things
MAD	median absolute deviation
KICT	Korea Institute of Civil Engineering and Building Technology
ARI	adjusted Rand index
NMI	normalized mutual information
DBSCAN	density-based spatial clustering of applications with noise
2D	two-dimensional
BCV	between-class variance
LOSO	leave-one-site-out
LOEO	leave-one-event-out
CI	confidence interval
FNR	false-negative rate
PCA	principal component analysis
t-SNE	t-distributed stochastic neighbor embedding
LDA	linear discriminant analysis
AD, MF, OT	Actual Displacement, Malfunction, Others
